# The Venom Gland Transcriptome of *Latrodectus tredecimguttatus* Revealed by Deep Sequencing and cDNA Library Analysis

**DOI:** 10.1371/journal.pone.0081357

**Published:** 2013-11-28

**Authors:** Quanze He, Zhigui Duan, Ying Yu, Zhen Liu, Zhonghua Liu, Songping Liang

**Affiliations:** 1 Cooperative innovation center of engineering and new products for developmental biology, College of Life Sciences, Hunan Normal University, Changsha, P. R. China; 2 The State Key Laboratory of Genetic Engineering, Institute of Biomedical Science, Fudan University, Shanghai, China; The City University of New York-Graduate Center, United States of America

## Abstract

*Latrodectus tredecimguttatus*, commonly known as black widow spider, is well known for its dangerous bite. Although its venom has been characterized extensively, some fundamental questions about its molecular composition remain unanswered. The limited transcriptome and genome data available prevent further understanding of spider venom at the molecular level. In the present study, we combined next-generation sequencing and conventional DNA sequencing to construct a venom gland transcriptome of the spider *L. tredecimguttatus*, which resulted in the identification of 9,666 and 480 high-confidence proteins among 34,334 de novo sequences and 1,024 cDNA sequences, respectively, by assembly, translation, filtering, quantification and annotation. Extensive functional analyses of these proteins indicated that mRNAs involved in RNA transport and spliceosome, protein translation, processing and transport were highly enriched in the venom gland, which is consistent with the specific function of venom glands, namely the production of toxins. Furthermore, we identified 146 toxin-like proteins forming 12 families, including 6 new families in this spider in which α-LTX-Lt1a family2 is firstly identified as a subfamily of α-LTX-Lt1a family. The toxins were classified according to their bioactivities into five categories that functioned in a coordinate way. Few ion channels were expressed in venom gland cells, suggesting a possible mechanism of protection from the attack of their own toxins. The present study provides a gland transcriptome profile and extends our understanding of the toxinome of spiders and coordination mechanism for toxin production in protein expression quantity.

## Introduction

Spiders are the largest population of venomous organisms [1] and they have been in existence for more than 300 million years, since the first true spiders (thin-waisted arachnids) evolved from crab-like chelicerate ancestors [2]. As ancient creatures, spiders are invaluable model organisms for research in evolution, ecology and medicine. As active hunters, they paralyze and kill prey by injecting venoms intended for hunting or defense. Spider venoms are complex mixtures consisting of a large number of toxins with distinct functions. Several toxins have been used as molecular probes, in particular in studies on ion channels and neurological disorders [3-5]. In recent years, an increasing number of spider toxins have been considered as potential drugs for the treatment of neurological diseases [6,7]. However, our understanding of spiders and their venoms is limited. This can partly be attributed to the lack of genome and transcriptome data on spiders. At present, no spider genome sequencing has been reported and the transcriptomic analyses of the venom glands of only ten spider species have been carried out; given nearly 40,000 spider species exist in the world, only a small number (about 37222)of nucleotide sequences have been deposited (NCBI Nucleotide database, August 31, 2013), which seriously limits research on the nature of spider venoms.

In recent years, the development of next-generation sequencing technology has greatly improved the sensitivity and efficacy and decreased the cost of sequencing. Next-generation sequencing has therefore been widely used in genomic and transcriptomic analyses [8-14]. Furthermore, the optimization of *de novo* sequence assembly algorithms for deep sequencing have enabled the accurate assembly of fragment data from sequencing into full-length transcripts [15]. These technologies have been widely used/validated in large-scale genomics and transcriptomics sequencing projects, in particular in the absence of a reference genome sequence [16]. 

**Figure 1:**
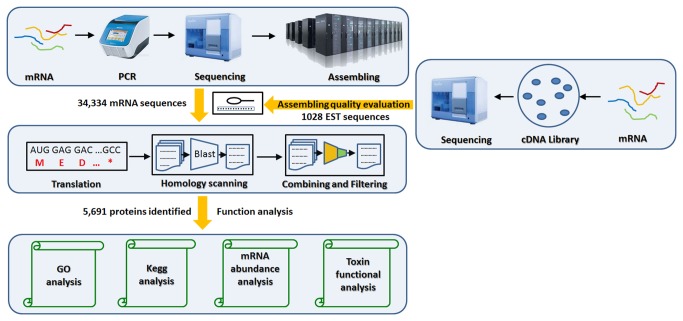
The pipeline of data process and analysis.

Here, we present the first transcriptomic profile of the venom gland of the spider *L. tredecimguttatus* obtained by combining cDNA library sequencing and next-generation sequencing (Illumina) coupled with *de novo* assembly (Figure 1). It includes 10,379 transcripts encoding 9,666 high-confidence proteins and 146 toxins. Our bioinformatics analysis revealed functional relationships between them and identified characteristics specific to the venom gland transcriptome, which broadens our understanding of spider venom composition and the cellular metabolism of the spider venom gland. Our findings suggest that deep sequencing coupled with *de novo* assembly is a powerful method for the study of the transcriptome of organisms lacking a reference genomic sequence. 

## Results

### cDNA library sequencing

Conventional cDNA library sequencing was first used to generate a small but highly confident dataset, which was also used for quality control of the data obtained by high-throughput *de novo* sequencing (see materials and methods). As a result, 1,015 unique EST sequences were identified and translated into 1,238 proteins. Of these, 480 were homologous to sequences in the Uniprot database (Magrane and Consortium 2011) based on a BLASTpx search (e-value less than e10-5) and 402 were high-confidence proteins (ML/BPL > 0.5, ML/PL > 0.5 and identity > 50%). Among these 402 proteins, 263 proteins are identified as toxin-like proteins. They could be classified into eight superfamilies, of which five superfamilies are homologous to five known toxins of *Latrodectus tredecimguttatus* (Swissprot ID: P23631, Q25338, Q02989, Q4U4N3 and P49125), respectively, and three superfamilies show high homology with known wolf spider (*Lycosa singoriensis*) toxins (Swissprot ID: B6DCN9, B6DCT8, B6DD16P84033), respectively. The gene ontology (GO) analysis indicated that 268 out of 480 proteins were located on the extracellular region as neuropeptide hormones (Figure S1 in File S1). A total of 65 unique full-length proteins were identified after filtering with stringent criteria (ML/BL and ML/PL > 0.5 and identity > 80%, please see details in the methods section).

### Deep sequencing and *de novo* assembly

High-throughput paired-end RNA-sequencing was performed on the cDNAs from poly (A)-enriched RNAs extracted from six venom glands of three mature spiders (*Latrodectus tredecimguttatus*) by using Illumina. Base the positive correlation between variant calling sensitivity and increased read depth in previous studies[17] (http://cdn.intechopen.com/pdfs/22515/InTech-Deep_sequencing_data_analysis_challenges_and_solutions.pdf), we increased the number of sequencing cycles for achievement high sensitivity and coverage with low copy transcripts and retrieved 4.7 GB of raw data containing more than 27 million 90 bp paired-end reads after removing low quality ones. The average sequencing depth was approximately 24 by compare predicting result of spider genome size (approximately 1.9 Gb)[18] and an empirical percentage (5%) of transcriptome size with genome size in the *Latrodectus* family as a reference [18,19] (see method). The raw sequencing data and assembled sequences can be downloaded from SRA and TSA of NCBI using accession numbers SRX337503 and GANL00000000, respectively.

All reads were assembled by the software Trinity [20] with default parameters, which generated 34,334 unique transcripts with a length of > 200 bp (Figure S2 in File S1), among which 1,321 transcripts were > 2000 bp. The mean length was 628 bp. Based on the resolving of Trinity assemble result, 9,094 transcripts shared common fragments (among 9,094 transcripts, two or more shared common fragments) and could be clustered into 3464 groups. The remaining 25,240 transcripts were distinct singletons (Table 1) that did not share any fragment.

**Table 1:** Statistics of RNA-sequencing and assembly results.

Total number of pair-end reads	27,605,467
Number of base pairs (bp)	2,484,492,030
Average length of reads (bp)	90
Number of transcripts	34,334
Mean length of transcripts	628 bp
Number of transcripts more than 2000 bp in length	1,312
Unique clusters	9,094
Distinct singletons	25,240

### Deep sequencing core dataset

As each transcript had six possible reading frames and could be translated into six amino acid sequences, we translated 27,453 cDNA sequences to all potential translation products (amino acid sequences) as candidates. Based on the length of sequences and their sequence similarity to known protein, the best translation product is determined for each transcript if there is. Firstly, sequences shorter than 40 amino acids were removed. And then, remained candidates were BLAST [21] against the Uniprot database [22]. The longest candidate with any homologues (e-values < e^10-5^) was considered as the best match. If there are no homologues founded, we just choose the longest one. Finally, we combined redundancy sequences, which match the same known protein and created a protein list containing 9,666 unique protein sequences (5,395 full length sequences and 4,271 fragments) as the high confidence core dataset. 

**Figure 2:**
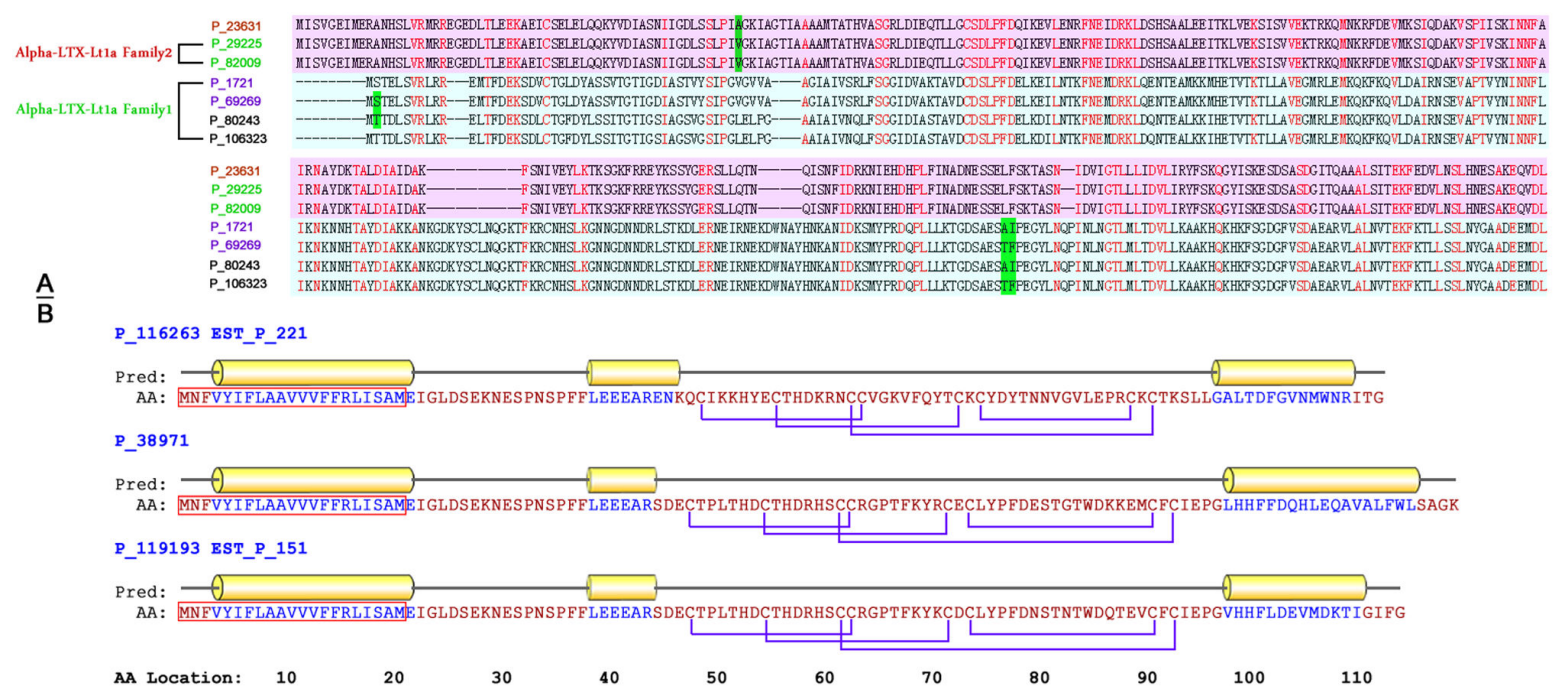
Examples of new toxins. A) Sequence alignment of seven members of the α-LTX-Ltla-1/2 families. P23631 is the Uniprot ID for α-LTX-Ltla-2, a well-known toxin of *Latrodectus* *tredecimguttatus*. The other six proteins are new potential toxins found in our core dataset. Amino acid residue point mutations are marked in green; Residues conserved across two families are marked in red. The two families are indicated by pink and cyan backgrounds. B) The secondary structure of three new toxins (P_206187, EST_P_221, P_141871, EST_P_151 and P_208737, members of the ctenitoxin family) is shown. The amino acids forming an alpha helix are colored in blue; red rectangles indicate predicted signal peptides; purple lines represent disulfide bridges.

In the core dataset, we identified the six previously reported toxins of the spider *L. tredecimguttatus* (Swissprot ID: P23631, Q25338, Q9XZC0, Q02989, P49125 and Q4U4N3 ) [23-28] and homologues of 14 toxins from other spider species. Specifically, all members of the histone family and a novel toxin family (α-LTX-Lt1a Family1) were also included in the dataset (Figure 2A and B), indicating the success of the strategy.

### Quality control of the core dataset

We developed a strategy to evaluate the quality of the core dataset by comparing it with known sequences from the cDNA library at the transcriptome level and Uniprot database at the proteome level. The basic principle of this strategy was that the probability of existence of known homologues is higher in databases of correctly assembled sequences than in those of wrongly assembled sequences. We defined several parameters to evaluate the similarity of a bait protein to its homologues (prey) including length of the matched region between bait and prey (ML), length of the bait sequence (BL), length of the prey sequences (PL), and the identity ratio between bait and prey sequences (identity). The values of ML/BL, ML/PL, and identity were used to evaluate assembly accuracy, sequence integrity and variation, respectively. 

**Figure 3:**
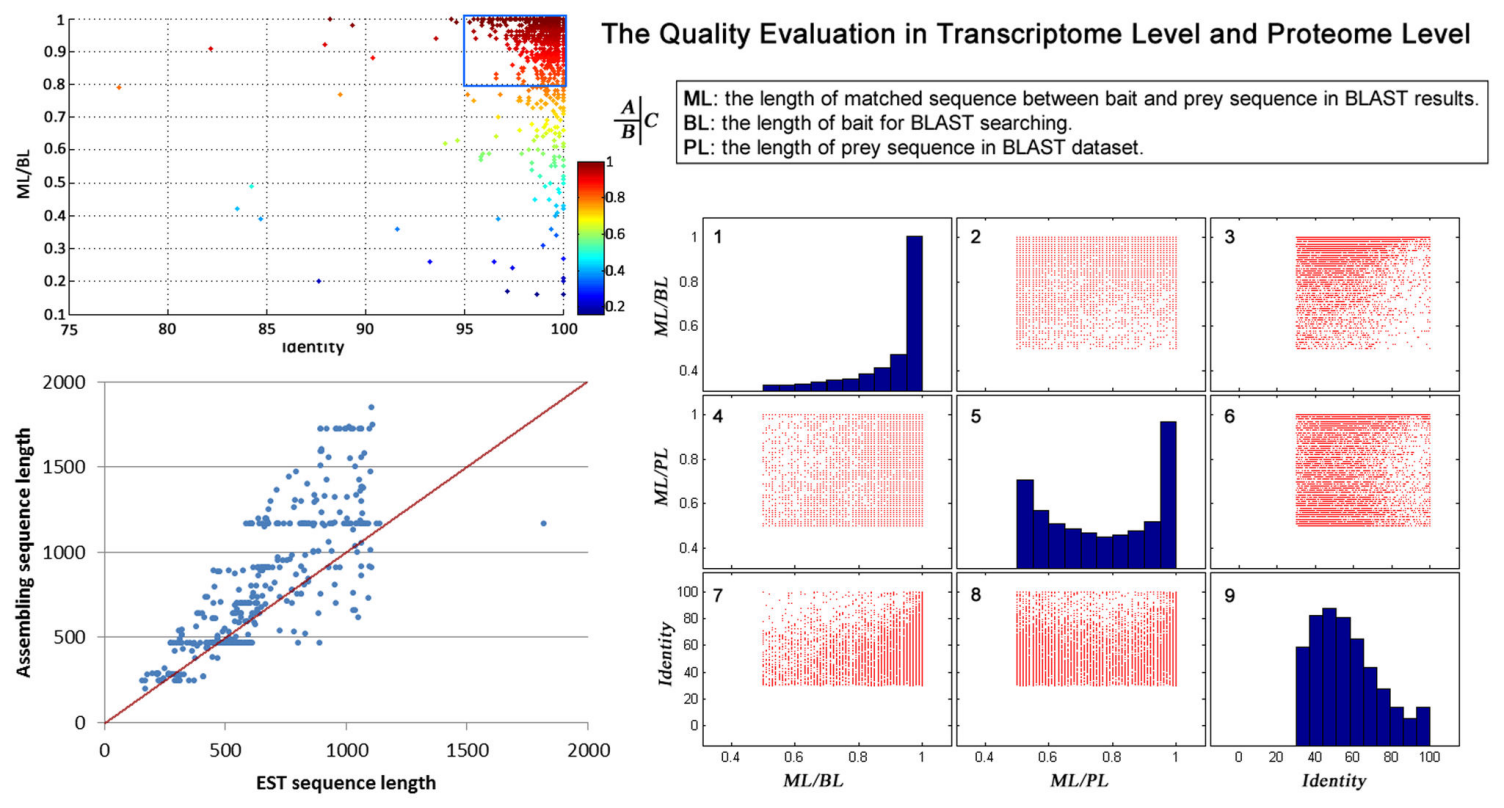
Statistical analysis of BLAST searches. A) Dot plot of ML/BL vs. identity of the BLAST queries, which searched against known spider EST datasets with assembled cDNA sequences (see methods). B) Length distribution of transcripts shared by the *de* *novo* assembly and EST sequencing datasets. C) Statistical analysis of protein homologues identified by a BLAST search against the Uniprot database performed with all translated protein sequences. Figures C1, C5 and C9 are bar charts for ML/BL, ML/PL and identity distribution. Other dot plots represent the pairwise correlations among them.

At the transcriptome level, the assembled sequences were blasted against the cDNA library sequencing data (EST sequences) of the spider *L tredecimguttatus*. As a result, 961 out of 1,015 (94%) EST sequences matched the assembled sequences with e values < 10^-5^, and 824 out of 961 (≈90%) EST sequences shared >80% sequence coverage and > 95% sequence identity with the assembled sequences (Figure 3A), Reverse EST sequences were used as negative controls. No significant matches were detected between negative control and assembled sequences, indicating a low false positive ratio and high quality of our assembled core dataset. 

The full sequence length is another important character for evaluating sequence quality in de novo sequencing [20,29]. In the present study, we compared the length of 620 EST sequences and their homologues in the core dataset (both ML/BL and ML/PL > 0.5). Our results showed that 442 out of 620 EST sequences were shorter than their homologues, indicating that *de novo* DNA assembly had the advantage of accessing full length transcripts (Figure 3B). 

At the protein level, 9,666 high-confidence protein sequences in the core dataset showed high matching coverage (both ML/BL and ML/PL > 50% and identity > 30%) with the corresponding homologous sequences in the Uniprot database, with 2,699 protein sequences showing both ML/BL and ML/PL > 80% and sequence identity > 50% (Figure 3C). As shown in Figure 3C1, 5 and 9, the distribution of the ML/BL and ML/PL of identity differed significantly in the core dataset. No positive correlations between identity and ML/BL and ML/PL (Figure 3C6) were observed, suggesting that sequence variation was derived from species evolution but not from error of sequence assembly. 

### Abundance of transcripts

Reads per kilobase of exon model per million mapped reads (RPKM) is a method to quantify gene expression [8]. In the present study, a Perl script was used to calculate RPKM for each assembled cDNA sequence with parsing alignment data using the software Bowtie [30]. The sum of the RPKMs of 9,666 high-confidence transcripts was 762,905.1, with a mean value of 78.92. The dynamic range of RPKM in the venom gland transcriptome was >10^6^ in amplitude. Recent studies have suggested a high correlation between the abundance of transcripts and proteins [31]; therefore, the RPKMs of transcripts were used to elucidate the abundance of the corresponding proteins. Sorting RPKM indicated that an α -LTX-associated low molecular weight protein (LMWP, P_89055) had the highest transcript expression level (216,786) and the remaining four toxins (P_86431, P_119193, P_116263, P_115505) in the top twenty highly expressed transcripts.

### GO annotation and analysis

**Figure 4:**
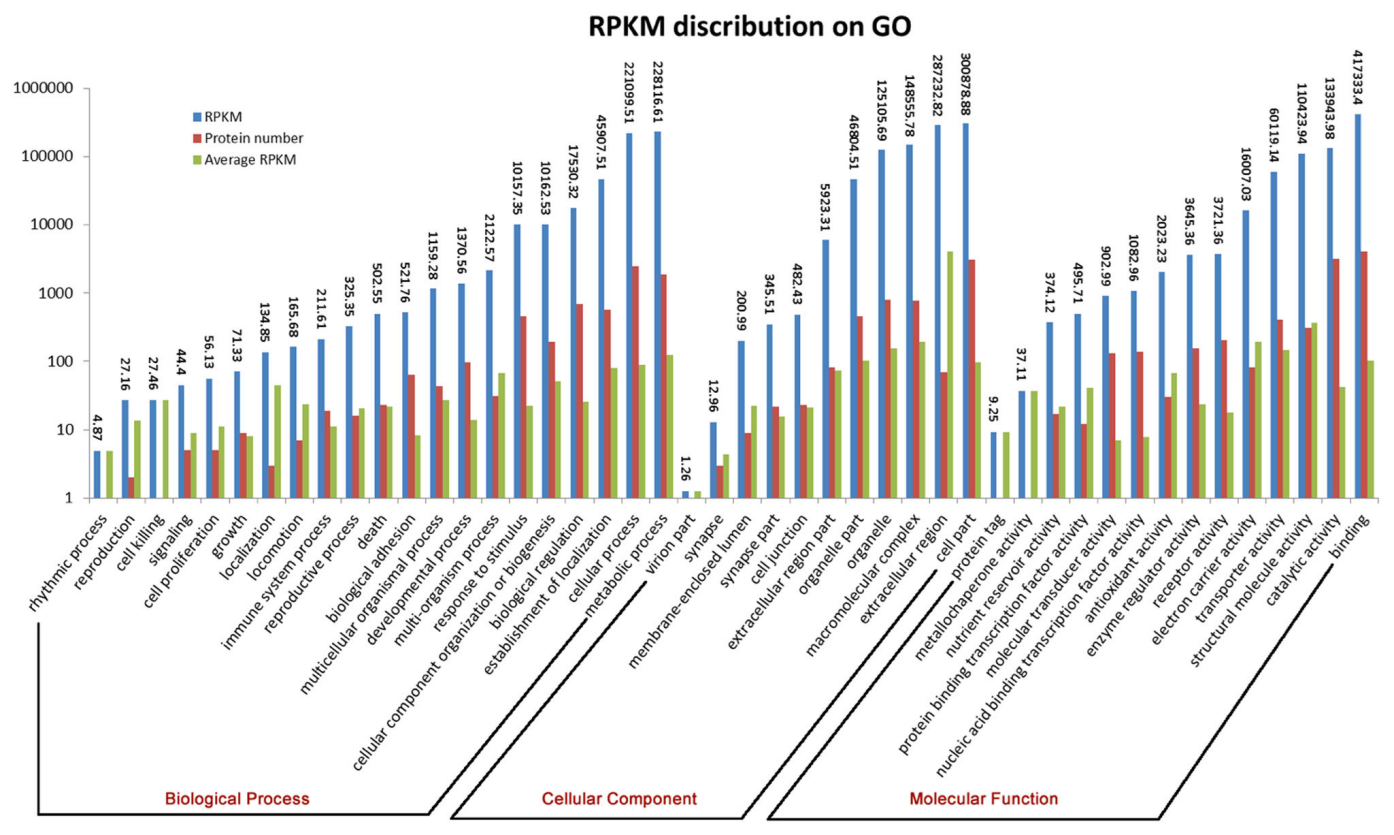
The RPKM distribution of transcripts in different categories and three GO namespaces. For each GO category, the sum of RPKMs, protein number and RPKM average were calculated and shown as blue, red and green bars, respectively.

To explore the functional characteristics of the transcriptome, we elucidated the functions of transcripts on the basis of GO annotations of their homologues. Overall, 6,191 of 9,666 high-confidence proteins were linked with GO annotations and were classified into 46 subgroups within three namespaces of GO, namely biological process, cellular component and molecular function (Figure 4). Statistical analyses indicated that some GO terms that may be important for venom gland function were highly enriched in the venom gland transcriptome. The RPKMs significantly enriched two GO terms “extracellular region” and “cellular component”; 69 transcripts were annotated accordingly. Four transcripts encoding four proteins (P_42039-F, P_89055, P_38395-F, and P_66861) were identified with the function “neuropeptide hormone” based on GO molecular function annotations, which is consistent with the main function of the venom gland in producing and secreting venom (Table S1 in File S1). Additionally, many transcripts/proteins involved in protein metabolism, including translation, transportation, energy metabolism, and post-translational modifications were highly expressed in venom gland cells, indicating active metabolic processes necessary for generating enough energy and materials for fast toxin production (Table S1 in File S1). For example, disulfide isomerases (P_49789, P_137-F, P_35521, P_131233, P_13411, P_13935-F ), which play important roles in the formation of correct scaffolds by catalyzing disulfide bond formation between two cysteines in toxin proteins, were highly expressed [32]. Other examples were provided by two proteins (P_7389 and P_86431) with high RPKM (11,481.33−145 fold and 8632.21−109 fold higher than the mean RPKM). P_7389 is a homolog of proteolysis enzyme E0W1I2_PEDHC (Swissprot ID), an intracellular protein involved in proteolytic processing into biologically active peptides [2]. The protein P_86431, as a homologue of U21-ctenitoxin-Pn1a, is also an enzyme involved in proteolysis but it is located in the extracellular region. Although the detailed functions of these proteins remain the subject of further investigation, it is attractive to speculate that P_7389 may play a role in toxin maturation and P_86431may be a high abundance component of the venom responsible for tissue digestion in the prey 

One of the interesting questions about venom gland cells is how they protect themselves from the attack of the toxins that they generate. Several studies have indicated that venomous animals such as scorpions are also sensitive to their own toxins [33,34]. The ion channels in the nervous system of some venomous animals have been shown to acquire resistance to their own toxins through mutations in key amino acid residues in the nervous system [33]. Voltage-gated ion channels, such as voltage-gated sodium, potassium and calcium channels, are the main targets of most toxins from spider venoms. Therefore, we analyzed the expression of these ion channels in the venom gland and found a voltage-gated calcium channel subunit and a putative potassium channel in our core dataset that were expressed at low levels (8.81 and 14.34); other voltage-gated sodium, potassium and calcium channels and α -LTX receptors were not detected in venom glands (Table S2 in File S1). The results suggested that the tissue-specific absence of toxin targets on the surface of venom gland cells makes them immune to toxins.

### KEGG annotation and analysis

Pathways that are important for venom gland function were investigated by searching the KEGG database using all protein sequences in the core dataset, which showed that 1,455 proteins were distributed in 164 KEGG pathways divided into six classes (cellular processes, environmental information processing, genetic information processing, human diseases, metabolism and organismal systems) (Figure S3 in File S1). Accumulation proteins RPKM of each pathway, respectively, we observed that 21 genetic information processing pathways occupied 48% of all 164 pathways (Table S3 in File S1). More than 50% of these 21 genetic information processing pathways were associated with transcription, protein production and transportation processes (Table S4 in File S1), such as ribosomes, spliceosomes, protein export, and protein processing in the endoplasmic reticulum among others. A hypergeometric distribution test [35] was used to estimate pathways enriched in these transcripts (Table 2), which showed that six of the top ten enrichment pathways were related to protein production and the others were associated with metabolism and digestion (Figures S4 and S5 in File S1). This observation is consistent with the GO analysis showing that metabolic pathways are highly active in venom gland cells. 

**Table 2:** The top ten enriched KEGG pathways of the spider venom gland transcripteome.

**KEGG Pathway Name**	**Pathway Classification**	**RPKM**	**P value**
Protein processing in endoplasmic reticulum	Folding, Sorting and Degradation	5193.12	5.65366E-31
RNA transport	Translation	4974.49	3.56164E-22
Spliceosome	Transcription	1183.85	3.93501E-21
N-Glycan biosynthesis	Glycan Biosynthesis and Metabolism	713.32	2.21443E-11
Ubiquitin mediated proteolysis	Folding, Sorting and Degradation	1168.3	5.98339E-11
Ribosome biogenesis in eukaryotes	Translation	712.82	6.90752E-11
Basal transcription factors	Transcription	327.53	1.42435E-10
Lysosome	Transport and Catabolism	789.45	1.77229E-10
mRNA surveillance pathway	Translation	581.5	9.05468E-10
Endocytosis	Transport and Catabolism	1306.28	2.30193E-08
Peroxisome	Transport and Catabolism	622.53	3.29395E-07

### Toxinome of *Latrodectus tredecimguttatus*

**Figure 5:**
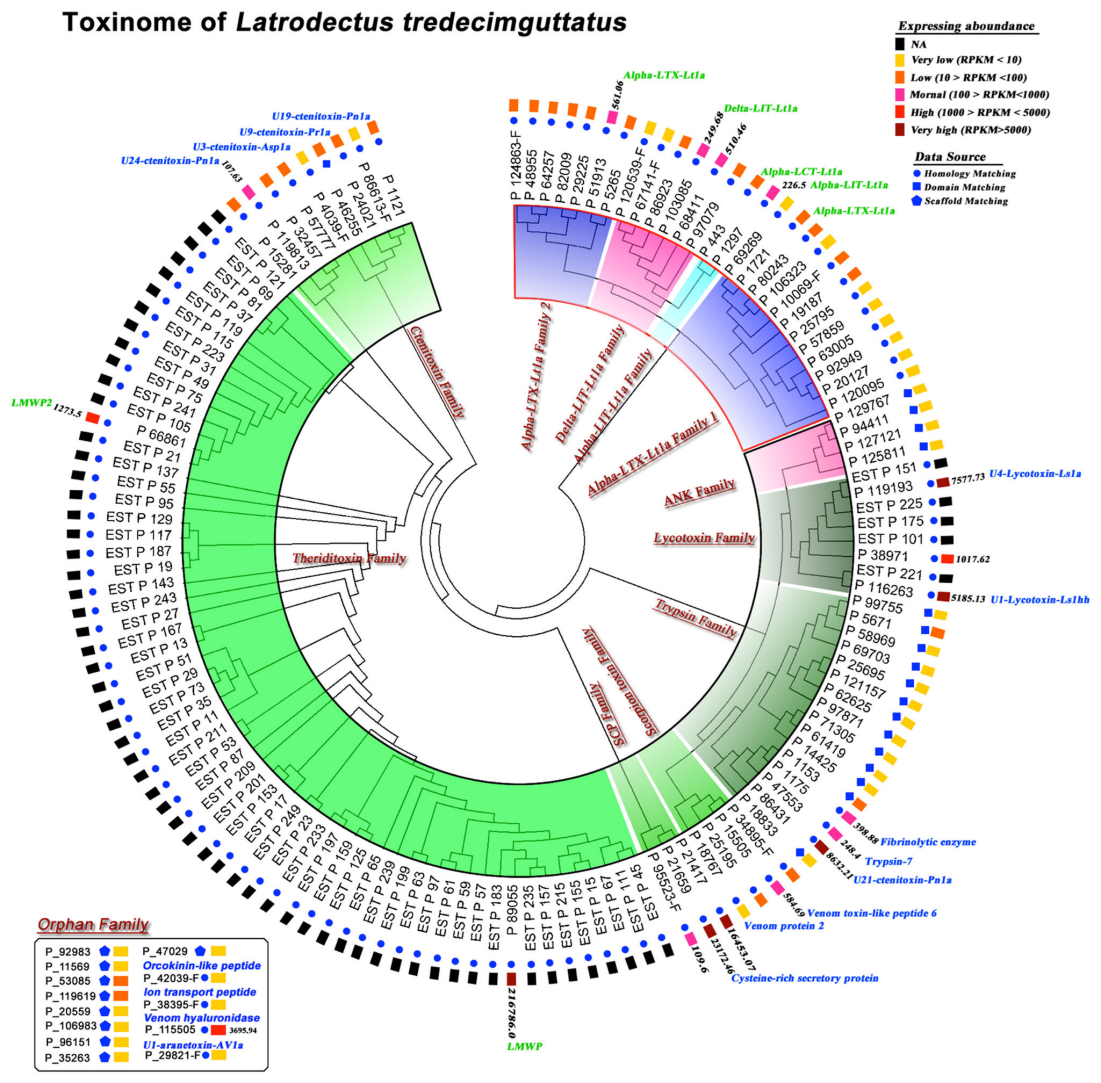
Cluster of the toxinome of *Latrodectus* *tredecimguttatus*.

**Figure 6:**
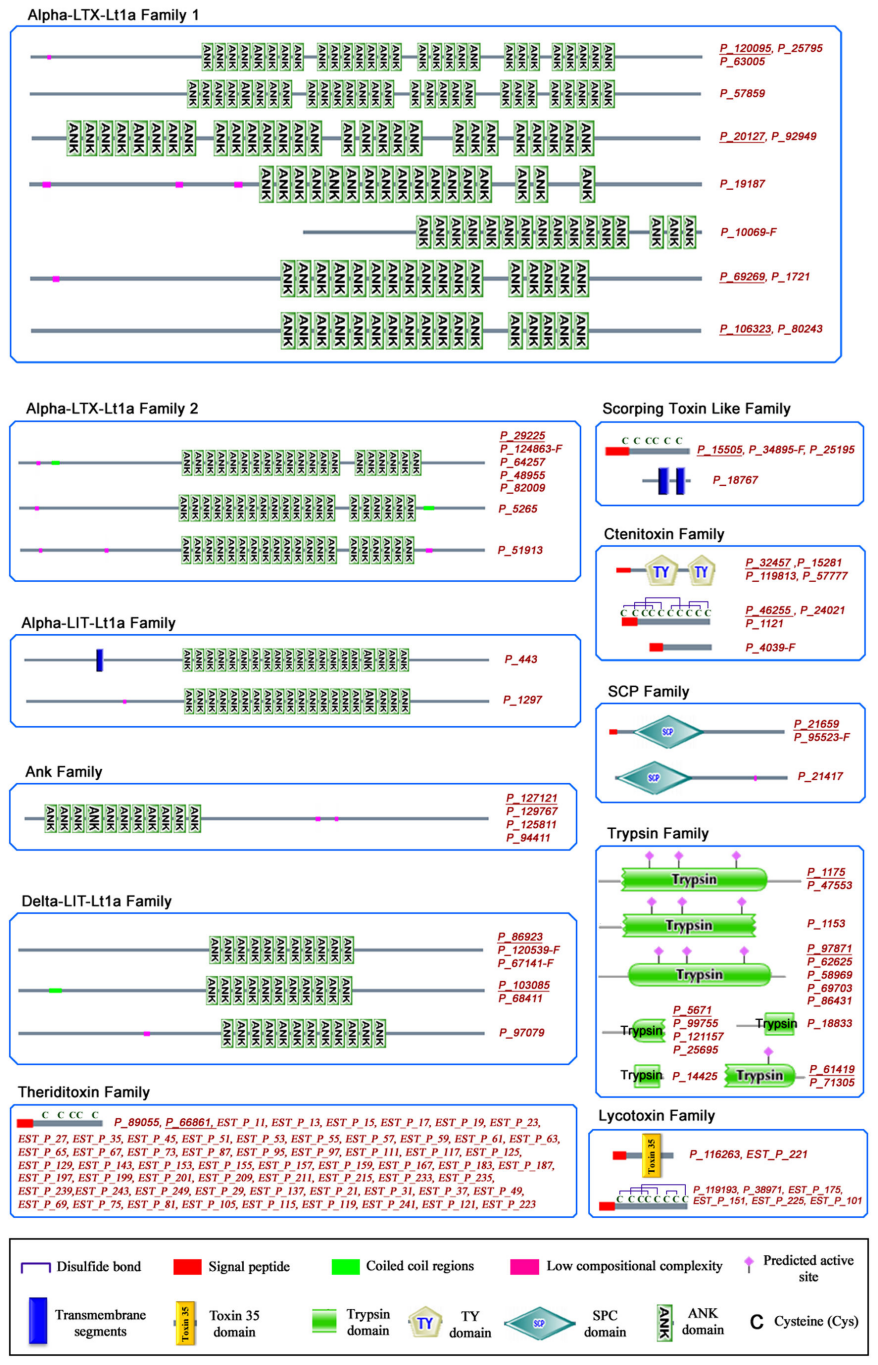
Domain architecture of toxins. Domain architectures of toxins were predicted by the SMART and Pfam servers (http://smart.embl.de and http://pfam.janelia.org) [59,60]. All toxins were grouped based on the respective family classification. The character “-F” appended to protein ID numbers indicates that these sequences are fragments and not a full-length protein. The character “EST_” appended to protein ID numbers indicates that these sequences were extracted from the EST dataset. Proteins sharing the same domain architecture were combined. The members of the Ank superfamily and related legends are listed in the middle figure. For the orphan family, multiple sequence alignments of potential toxins and known toxins (green labels) are shown and the matched cysteine domains are indicated. The abbreviations of domain names are as follows: ankyrin repeats (ANK, SMART ID: SM00248); TY (SMART ID: SM00211); trypsin (Pfam ID: PF00089); SCP (SMART ID: SM00198); EGF (SMART ID: SM00181); RHO (SMART ID: SM00174); toxin 35 (Pfam ID: PF10530).

One of main goals of the present study was to discover novel toxins. Three strategies were used to identify potential toxins: sequence homology searching, domain prediction and cysteine knot structure alignment (see methods and [39]). We identified 146 toxins of which 81 were derived from deep sequencing and 65 were from the EST dataset. The sum of the RPKMs of the 81 toxins was 291,982.02, which was equivalent to one third of the RPKM of the core dataset (PKRM = 762,905.1) (The RPKM of EST sequences was NA). These results were reasonable and consistent with the major function of the venom gland, namely the generation of toxins. Notably, all 6 known toxins from *Latrodectus tredecimguttatus* and homologues to 16 known toxins from other species (Table S5 in File S1) were included in our dataset. Of the 81 toxins identified by deep sequencing, 25 were identified by domain scanning or cysteine pattern alignment. Cluster analyses using ClustalX 2.1 in NJ model categorized them into the ANK superfamily and seven other families including trypsin, scorpion toxin-like, lycotoxin, ctenitoxin, theriditoxin, SCP and orphan families, which with the exception of theriditoxin, were all first reported in *Latrodectus tredecimguttatus* (Figure 5). The domain architectures of all families indicate the huge sequence as well as functional diversity (Figure 6).

The solid blue circles, squares and pentagons are markers for identification methods. The expression levels of transcripts are indicated by rectangles of different colors. All toxin families are labeled and highlighted with colorful backgrounds. The character “-F” appended to protein ID numbers indicates that these sequences are fragments and not full-length proteins. “EST” appended to protein ID numbers indicates that these sequences were identified from the EST dataset and RPKM is NA. RPKM values are shown for all toxins with high or very high expression levels. Green labels indicate known toxins in *Latrodectus tredecimguttatus* and toxin homologues of related transcripts; blue labels indicate firstly reported toxins in *Latrodectus tredecimguttatus* and toxin homologues of related transcripts. Data on the orphan family is shown in the top right box. Underlined family names represent novel toxin families

### ANK Superfamily

The ANK superfamily is characterized by the presence of several ANK domain repeats (ankyrin repeats, SMART ID: SM00248) and contains five families: α-LTX-Lt1a family 1, α-LTX-Lt1a family 2, α-LIT-Lt1a, δ-LIT-Lt1a and ANK family (Figure 6). The ANK domain is approximately 33 amino acids in length and its structure contains a helix-loop-helix. These families differ in the number and distribution of ANK domains and they are named after known toxins. For example, the α-LTX-Ltla family 2 includes toxins with 20 ANK domains located in the central part and they are split into two parts as a 14+6 pattern. For the α-LIT-Lt1a and δ-LIT-Lt1a families, consecutive ANK domains are located on the central part of the toxins, whereas diverse patterns and more ANK domains are found in the toxins of the α-LTX-Lt1a Family 1. Consecutive repeats of ANK domains have been shown to be involved in protein-protein interactions and may direct their binding to receptors [40]. ANK domain repeats in the central and C-terminal regions were reported to be very important for pore formation through the assembly of tetramer complexes for the induction of exhaustive neurotransmitter release in vertebrates [41,42]. Some well-known toxins in these families, such as α-LTX-Lt1a and α-LIT-Lt1a, were shown to be the main neurotoxins responsible for human envenomation syndrome by specifically binding neuronal cell receptors on the presynaptic plasma membrane. δ-LIT-Lt1a, which lacks C-terminal ANK domain repeats, was also reported to induce exhaustive neurotransmitter release in insects, but not in vertebrates, through a similar mechanism [42]. All four key residues (C34, C91, C413 and L448) of α-LTX-Lt1a toxin are conserved in α-LTX-Lt1a family 2. Interestingly, with the exception of L448 in α-LTX-Lt1a family 1, the other three key amino acids are absent in the δ-LTX-Lt1a family, suggesting that the toxin functions of the δ-LTX-Lt1a family may be different from those of α-LTX-Lt1a family 1 and 2. Additionally, the various architectures based on the ANK domain in this superfamily suggest that the genes encoding these toxins may undergo active duplication to generate diverse functions, indicating that they undergo a strong positive selection pressure. On the basis of abundance analysis, we discovered similar toxins expressed at higher levels that could represent potentially important toxins, although their function remains unclear. For example P_51913, a member of the α-LTX-Lt1a family, has a similar length and domain architecture but much higher expression level than other members of the α-LTX-Lt1a family 2. 

### SCP Family

The SCP family includes three members (P_21417, P_21659and P_95523-F) and is characterized by the SCP domain. SCP (SMART ID: SM00198) domain (Figure 6), a cysteine-rich motif generally found in secretory proteins that plays a role in the construction of the extracellular matrix, branching morphogenesis and ion channel regulation in fertility [43]. Proteins containing an SCP domain, which are present in venoms from various arthropods including wasps, fire ants, scorpions (Swissprot ID: C5J8B3) and spiders (Swissprot ID: A9QQ26 ) (*e.g. Lycosa singoriensis*), have the ability to inhibit ryanodine receptors, a class of calcium-induced calcium release channels found in animal muscles and neurons [44]. The widespread existence of these toxins in different venomous arthropods indicates that they may be a class of ancient toxins inherited from a common arthropod ancestor.

### Ctenitoxin Family

The ctenitoxin family (ctenitoxins) was first identified in the venom of *Latrodectus tredecimguttatus* and later detected in the venom of wolf spiders. According to our data, this family includes 9 members with three architectures: two consecutive TY domains (SMART ID: SM0021), a shared cysteine rich-pattern and an uncharacterized scaffold. Each of four toxins （P_32457, P_15281, P_119813 and P_57777,) contains two TY domains (SMART ID: SM0021) and a predicted signal peptide in its sequence (Figure 6). The same TY domain architecture has been found in spider toxins such as B5M6G6 (Swissprot ID) (from *Ornithoctonus huwena*) and P84032 (Swissprot ID) (from *Phoneutria nigriventer*). TY domain-containing proteins including P84032 have been proposed to be cysteine proteinase inhibitors [45], suggesting that these four toxins may function as proteinase inhibitors. The other four members (P_46255, P_24021, P_1121 and P_4039-F) are homologues of known ctenitoxin toxins (U19-CNTX-Pn1a, U9-CNTX-Pr1a, U3-AATX-Ce1a and U3-CNTX-Asp1a) from the venom of *Phoneutria nigriventer*, *Phoneutria reidyi*r, *Caerostris extrusa* and *Ancylometes* *sp*, respectively. Of the four toxins, P_46255, P_24021 and P_1121 share a cysteine-rich domain, whereas P_4039-F does not contain any known domain in its sequence.

### Lycotoxin Family

The lycotoxin toxin family was first discovered in *Latrodectus tredecimguttatus*. Of the eight members identified in this study, three toxins were identified in the core dataset (P_116263, P_119193 and P_38971) and another five were from the EST dataset (EST_P_255, EST_P_175, EST_P_101, EST_P_221 and EST_P_151) (Figure 6). They are homologous with U1-lycotoxin-Ls1hh and U4-lycotoxin-Ls1a from wolf spider venoms. They share a characteristic ICK-like motif (Toxin-35 domain) and may function as neurotoxins through their activity on ion channels [46]. Sequence analysis indicated that P_116263 and P_119193 are identical to EST_P_221 and EST_P_151 in amino acid sequence but differ slightly in their nucleic acid sequences. Domain and secondary structure prediction suggested that three toxins (P_116263, P_119193 and P_38971) share the same protein structure characterized by a highly conserved N-terminal including a predicted signal peptide, three α-helix regions with eight highly conserved cysteines and an ICK motif located in the center of the sequence (Figure 2C). However, the low sequence similarity between P_116263 and the other two members suggests that they have a long evolutionary history and possibly different functions. Furthermore, all members were highly abundant. The RPKMs of P_119193, P_116263 were found to be > 5000 and those of P_38971 were > 1000, indicating their functional importance. The sequence of P_119193 was similar to that of CSTX-1, an inhibitor of calcium voltage-gated channels [46]. This implies that these three toxins may function in a similar manner to block neuronal signal transduction by interacting with ion channels. 

### Theriditoxin Family

This family includes 62 members of which 14 toxin-like proteins are homologous with LMWP2 and the remaining proteins are homologous with LMWP. Sixty members were identified by conventional cDNA library sequencing and two [P_89055 (Swissprot ID: P49125) and P_66861 (Swissprot ID: Q4U4N3)] were derived from *de novo* deep sequencing data (Figure 6). Multiple sequence alignment indicated the presence of point mutations in this family that were also detected in other families and spider species [47], indicating a high evolutionary speed and strong positive Darwinian selection pressure. Secondary structure analysis showed that they share a cysteine-rich motif, and functional prediction indicated that they might act as assistant catalyzers, which may improve the toxicity of α-latrotoxin or other venom components [48]. Quantitative analyses indicated that LMWP was the most abundant toxin in the venom and its RPKM value was approximately 120 fold higher than that of LMWP2, highlighting the predominant role of LMWP in the spider venom and only exists in Latrodectus by Blast uniprot database. 

### Scorpion toxin like family

The family includes four members (P_15505, P_34895-F, P_25195 and P_18767) with homology to venom toxin-like peptide-6 and venom protein-2 from scorpion venom [49]. A cysteine-rich pattern characterized by six highly conserved cysteines is shared by P_15505, P_34895-F and P_25195. P_18767 contains two transmembrane segments and is expressed at a low level (Figure 6). Their functions are largely unknown.

### Trypsin Family

The trypsin family includes 16 members and all of them have a complete or partial trypsin domain (Pfam ID: PF00089). Trypsin domain-containing proteins are usually considered as potential hydrolases. Trypsin domain-containing toxins are widely distributed among spider (*Agelenidae*, *Ctenidae* and *Lycosidae*), scorpion and snake venoms [50]. Based on sequence alignment, five proteins (P_97871, P_62625, P_58969, P_69703 and P_86431) (Figure 6), as the representative toxins of the family, were homologous to B7QB06 from the venom of *Ixodes scapularis* [51] as proclotting enzyme. The high RPKM (8632.21) for P_86431 indicated that it may be expressed at a very high level. It may play important roles in toxin maturation and/or the hydrolysis of prey tissues [51]. The other two proteins (P_47553, P_1175) were also highly expressed and showed homology to U21-ctenitoxin-Pn1a of *Phoneutria nigriventer* spider [52] , indicating that they may play an important role in the venom.

### Orphan Family

In this family, the identification and classification of members are mainly based on the cystine knot pattern shown in Figure S6 in File S1. Nine out of 13 proteins had low sequence similarity with known toxins and therefore lacked clear functional annotations, while the remaining four proteins were identified by homology matching. For example, P_29821-F is homologous with U1-aranctoxin-AV1a [53]. Domain analysis suggested that the protein includes an intact KU domain, which is a catalytic domain of serine proteases present in many venomous organisms, especially in the phylum *Arthropoda*. KU domain-containing toxins may be ancient toxins and play a role in the inhibition of trypsin or voltage-gated potassium channels. The cystine pattern may be considered as an important property of animal toxins, although it is neither necessary nor sufficient for toxin identification [54,55].

Taken together, our data provided a global and comprehensive perspective of the toxinome of *Latrodectus tredecimguttatus*. Functionally, all toxin families can be classified into five categories as follows: 1) neurotoxins, including the ANK superfamily, the SCP family and the lycotoxin family, which can interact with receptors or the cell membrane to directly interfere with the transmission of neural signals; 2) assistant toxins, such as the members of the theriditoxin family, which do not directly affect the targets of neurotoxins but assist and enhance their toxicities; 3) proteases, such as the proteins in the trypsin family that contribute to toxin maturation as well as prey tissue digestion; 4) protease inhibitors, such as TY domain-containing toxins in the ctenitoxin family, which can inhibit proteases and may play a role in protecting toxins from degradation; 5) unknown-function toxins including the members of the scorpion toxin like family and the orphan family. The abundance of all families and functional classification are shown in Figure S7 in File S1. 

Comparing our dataset with known toxinome of other venomous animals (Table S6 in File S1), we found one characteristic which distinct *Latrodectus tredecimguttatus* from other species is the lack of ion channel toxins. The ANK superfamily, main neurotoxin components of in venom, is very specific to the black widow spiders. They usually interact with receptor (such as G-protein coupled receptor) to regulate cytosolic concentration of IP3 and Ca^2+^ release but not target ion channels directly. And only one potential calcium voltage-gated channels toxin (P_119193) was discovered in the toxinome of *Latrodectus tredecimguttatus*. This support the hypothesis that spider widow spiders may be a kind of advanced spiders,

### Phylogenomic Analysis

**Figure 7:**
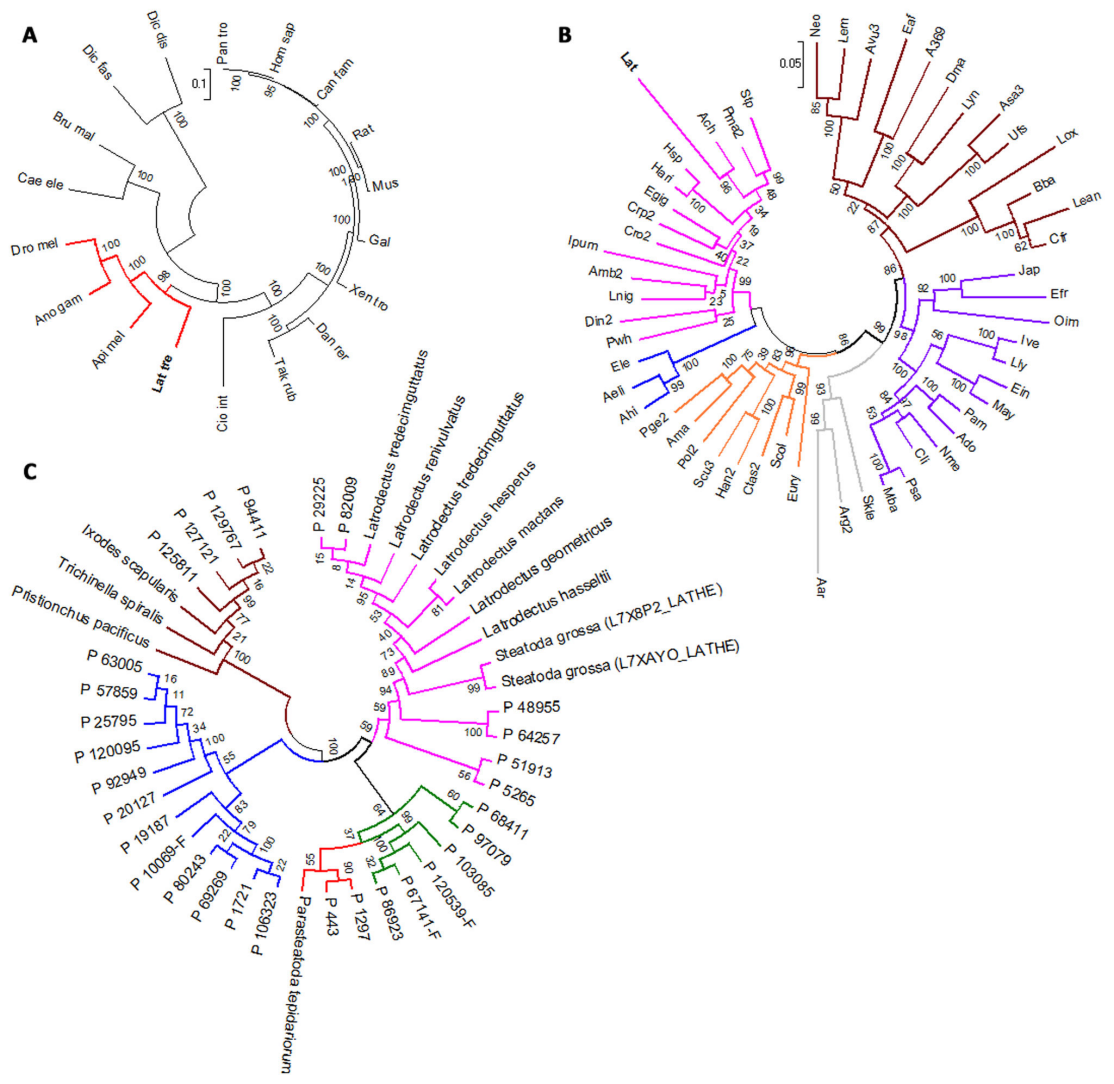
Evolutional relationship of species and toxins. A. Phylogenetic tree of the 26 species. It was constructed by MAGE software using sequences of single-copy nuclear protein-coding genes from these species. *Latrodectus* *tredecimguttatus* was grouped with other arthropods (insects) and highlighted in red. B. Phylogenies of Arthropod. *Latrodectus* *tredecimguttatus* was clustered with other three spider species (Theraphosidae, Amblypygi and Schizomidae) in Arachnida, which are marked with different colors: purple, Hexapoda; brown, Crustacea; silver gray, Oligostraca; orange, Myriapoda; blue, Pycnogonida; The full names of the species can be found in Tables S7 and S8 File S1. C. Phylogenies of the ANK superfamily, in which pink indicates α-LTX-Lt1a family1; blue indicates α-LTX-Lt1a family2; green indicates δ-LIT-Lt1a family; red indicates α-LIT-Lt1a family; brown indicates ANK family.

According to BLAST search, we observed that the homologous genes of toxins from *Latrodectus tredecimguttatus* are widely found in not only Arachnoidea but also in some more distant species such as fly, snake, wasps, and fire ants, indicating the high variety of origination and complex evolutionary processes of these toxins. To understand the evolutionary context of *Latrodectus tredecimguttatus*, we selected 66 protein sequences of single-copy nuclear protein-coding genes as baits to search homologues in Uniprot database [36] .All homologues are found from 54 arthropod species and 26 species from other taxonomic groups such as mammals, aves, amphibians, and arthropoda. They were aligned by ClustalX 2.1 and could be combined into one supergene for each species (see method and [37]). Finally, MEGA5.2 was used to remove low coverage sites (< 90%) among these supergenes and Maximum Likelihood Clustering analysis with 1000 bootstrap replicates were executed to generate phylogenetic tree (Figure 7A and B, Tables S7 and 8 in File S1). Two conclusions could be made from the result: (1) Hexapoda has a higher rank than Chelicerate and mammals are closer (Figure 7A) in terms of evolutionary rates which is consistent with the previous reports of Reiger and collaborators [36] and Martha and Alfredo [38]; (2) in the arthropod branch of the phylogenetic tree (Figure 7B), *Latrodectus tredecimguttatus*, *Aphonopelma chalcodes* (Ach; *Theraphosidae* family), *Phrynus marginemaculatus* (Pma2, Amblypygi) and *Stenochrus portoricensis* (Stp; Schizomida) were categorized into *Arachnoidea*, which s consistent with the known taxonomic classification of these organisms [38].

We also performed phylogenomic analyses for six newly discovered toxin families (trypsin, scorpion toxin-like, lycotoxin, ctenitoxin, theriditoxin, SCP) (Figure S8 and Table S9 in File S1) by BLAST search in Uniprot database and Maximum Likelihood Clustering analysis. We found that these toxin families have different originations and evolutional history. For example, the homologues of trypsin family toxins were found in fungi and other nonvenomous ancient animals, indicating they might be recruited from genes with normal physiological functions and develop new toxic functions in venomous species. On the other hand, lycotoxins may be originally a kind of native toxins because their homologues are only found in four closed spider species. It is reasonable to speculate that they are important to fulfill the specific demands of spiders to survive and the high abundances of them also highlight their functional significance. Additionally, phylogenetic analyses uncovered complex evolutionary relationship within toxin families. For ctenitoxin family, for example, P_32457, P_57777 and P_15281, were considered as original toxins because they have shortest distances to homologues from ancient animals. And P_1121 and P_24021 may be developed from them and was inherited by P_46255 and P_4039-F.

To investigate the evolutionary relationship within the five families in ANK superfamily, all sequences (31 toxin sequences) from this superfamily were BLAST against Uniprot database to search their homologues (p-value < 0.05). The result indicates that ANK toxin-like proteins (76 sequences) distribute widely in both invertebrate and vertebrate animals including 45 species, such as Hemichordate, Arthropoda, Actinopterygii, Amphibians, Aves, Mammal and so on. The phylogenomic tree constructed by MEGA 5.2 using Maximum Likelihood model with 1000 bootstrap replicates (see method) shows several interesting results (Figure S9 in File S1). First, among the five families, the ANK family was isolated from the other four families and has distinct architecture of ANK domains (Figure 6). Evolutionally, they clustered with homologues from parasitism arthropods that don’t have venom gland (such as *Ixodes scapularis* and *Pediculus humanus* *subsp*) and are closer to the homologues from other nonvenomous animals (such as frog, little brown bat, et al.) (Figure S9 in File S1, Figure 7C). Considering the aboundance of ANK family members are very low, it is reasonable to predict that they might not serve as captureing prey and have general functions which are similar with the homologous from other nonvenomous animals. Second, α-LTX-Lt1a family1 and their homologues are restricted in *Theridiidae* family (such as *Latrodectus Hesperus*, *Latrodectus tredecimguttatus*, *Steatoda grossa* and *Parasteatoda tepidariorum*), indicating these toxins might originate from the common ancient of *Theridiidae*. Third, no homologues of α-LIT-Lt1a family and α-LTX-Lt1a family 2 are found in Uniprot database, indicating that these families may be developed after the origination of the *Latrodectus tredecimguttatus* spider.

## Discussion

In the present study, conventional cDNA library sequencing and high-throughput sequencing combined with *de novo* sequence assembly were used to construct the venom gland transcriptome of *Latrodectus tredecimguttatus*. A comparison of the two methods identified clear differences: 1) deep sequencing provided a greater coverage of the transcriptome. In this work, a ten-fold higher number of transcripts were identified by deep sequencing than by cDNA library sequencing; 2) compared with cDNA library sequencing, the deep sequencing technology is more sensitive for detecting lowly expressed transcripts [8-14] . As shown in Figure 6, toxin families (α-LTX-Ltla, δ-LIT-Ltla, α-LIT-Ltla, α-LTX-Ltla 1, ctenitoxin and ANK families) expressed at relatively low levels were only discovered by deep sequencing but not by cDNA library sequencing; 3) conventional cDNA library sequencing may be more accurate for the detection of minor mutations among the members of a protein family, because it is difficult for *de novo* assembly algorithms to distinguish real mutations from sequencing error without a reference genome. Figure 6 shows paralogues of high abundance toxins identified by cDNA library sequencing such as P_89055 and P_66861; 4) because of the rapid development of *de novo* assembly algorithms, *de novo* deep sequencing is currently comparable, if not better, to EST sequencing for accessing full-length transcripts. As shown in Figure 2B, most of the assembled sequences were longer than those obtained by EST sequencing. Furthermore, updates in assembly algorithms have enabled the generation of more accurate transcriptome data without a reference genome sequences [56]. Therefore, the combination of next-generation sequencing and conventional cDNA library sequencing was shown to be an effective strategy for the construction of the venom gland transcriptome of the spider *L. tredecimguttatus*. The data from cDNA library sequencing also served as a reference to evaluate the quality control of *de novo* sequence assembly. Here, a core dataset containing 10,379 high confidence transcripts encoding 9,666 proteins, including 90 toxin-like proteins, was generated. This is the first report describing certain families (α-LTX-Lt1a family 1, trypsin family, lycotoxin family, SCP family, Ank family, scorpion like toxin family and ctenitoxin family) and toxins, and highly expressed toxins (e.g. P_89055, P_21417, P_21659, P_86431, P_119193, P_116263, P_95523-F, P_115505, P_66861 and P_38971), which are predicted to be functionally important, warrant further investigation. To our knowledge, this is the most comprehensive spider transcriptome /toxinome dataset reported to date. 

Functional and quantitative analyses of the venom gland transcriptome suggested the functional relevance and tissue specificity of gene expression. First, highly expressed transcripts were significant enriched in protein synthesis and metabolism related pathways, which are essential for toxin translation, transportation and secretion. Second, many extracellular and secreted proteins, especially toxins, were in high abundance. Third, most of the ion channels that may be potential targets of toxins were not expressed or expressed at low levels in venom gland cells, which may protect the venom gland cells from the attack of toxins. 

The venom is a complex mixture. We identified 146 toxin-like proteins forming 12 families that were categorized into five classes: neurotoxins, assistant toxins, proteases, protease inhibitors and unknown function toxins by deep sequencing and cDNA library analysis with venom gland of *Latrodectus tredecimguttatus*. Our data also reveal how these toxins work together: neurotoxins as the main weapons specifically target the nervous system to kill or paralyze prey; highly abundant assistant toxins may enhance the toxicity of neurotoxins by promoting the binding of neurotoxins to their targets; protease inhibitors may protect neurotoxins and assistant toxins from degradation by proteases; proteases may cleave precursors into mature toxins or aid in the digestion and consumption of prey. Three possible strategies were proposed for the prevention of toxin attack in venom gland cells: 1) low/non expression of toxin targets; 2) mutations of toxin targets; 3) inhibition of toxin maturation [23,26]. Our data support the first model that ion channels are usually non/lowly expressed in venom gland cells. 

In summary, our data and annotation pipeline not only presented us an overview of the cellular and molecular processes that take place in the venom gland of a spider but also identified new toxin families which could be considered as new pharmacological candidates for potential applications. However, It is should be noted that significant part of sequences (>30%) in our dataset are still functionally unknown. Extensive and intensive efforts for functional validation of these transcripts will be important for extending our understanding of the molecular complexities of spider venoms and their production in venom glands. .

## Methods

### Preparation, sequencing and assembly cDNA in *De Novo*

Total RNA was isolated from three *Latrodectus tredecimguttatus* venom glands using the TRIzol reagent (Invitrogen) and treated with RNase-free I. Poly (A) mRNA was isolated using oligo dT beads with random hexamer-primer and reverse transcriptase to synthesized cDNAs, and then digested with RNase H (Invitrogen). Finally, 200 bp DNA fragments were selected and prepared according to Illumina’s HiSeq 2000 protocols. A sequence of 90 bp from both ends of each fragment was determined. After filtering out lower quality short reads, the cDNA sequences were *de novo* assembled from two fastq files (include 27 million short reads) by the software Trinity [20] with default parameters in which the parameters “--jaccard_clip” has been used for split overlap UTR range of different transcripton. To estimate the average sequencing depth, we selected the largest known genome sequence (approximately 1.9 Gb) in the *Latrodectus* family as a reference [18,19] and divided the total length of the reads by the probable transcriptome size, which was 5% (human ~= 2.5%; Arabidopsis =~ 0.1%) of the largest genome size of the *Latrodectus* family (27*10^6^*90/1.9*10^9^*0.05 = 24) . 

### Constructing a cDNA library and sequencing

A directional full-length cDNA library was generated from the venom gland of *Latrodectus tredecimgattutas*. Fifteen L. *tredecimguttatus* female spider specimens were milked to stimulate the production of mRNAs in the venom glands. After 4 days, the venom glands (approximately 200 mg) of 15 individual spiders were isolated and immediately frozen in liquid nitrogen with grinding. The Trizol reagent (Invitrogen) was used according to the manufacturer's protocol for RNA extraction. The integrity of total RNA was checked by visualization of the 28S and 18S bands of ribosomal RNA in formaldehyde denaturing 1% agarose gels. The PCR-based cDNA library was constructed following the instructions included in the SMART cDNA library construction kit (Clontech, Palo Alto, CA, USA). Competent Escherichia coli 5Hα cells were transformed with the cDNA library plasmids to amplify the cDNA. The resulting colonies were randomly picked, and the inserted cDNAs in the individual colonies were directly amplified by colony PCR using universal M13 forward and reverse primer sets. The PCR products were resolved by agarose gel electrophoresis to determine the size of each product. Selected clones with 4400 bp cDNAs were analyzed with standard M13 forward primers on an ABI 3730 automatic DNA sequencer according to the manufacturer’s instructions (completed by Shanghai Sangon Biological Engineering Technology and Service Co. Ltd.)

### Translation and homologue searching

Transcripts were translated using a home-made Perl script for all possible ORFs following standard codons. Each assembly cDNA sequence was translated from a translation initiating codon to a stop codon. The proteins translated from cDNAs without a stop codon were categorized as protein fragments and the others were identified as full-length proteins. As a result, 65,669 unique potential amino acid sequences were obtained after filtering out sequences shorter than 40 aa. Homologue searching was performed by BLASTp querying against the Uniprot database with the threshold (E value cut-off) set at e^10-5^, which yielded 13,606 proteins with e-values < e^10-5^. For each protein, the ML/BL, ML/BL and identity were measured for the best match in the BLAST query, which identified 9,666 amino acid sequences with high ratios (ML/BL > 0.5, ML/PL > 0.5 and identity > 30) that were considered as high confidence proteins and were included in the core dataset. Finally, 6,191 high confidence proteins were identified.

### Hypergeometric statistical analysis

We used a hypergeometric test to identify KEGG pathways highly represented in the venom gland cell transcriptome. The KEGG database was downloaded on *2011/06/22*. All transcripts in the core dataset were analyzed by BLAST comparison against the KEGG database and mapped to KEGG pathways. All proteins in the core dataset were considered as a population (N). The transcripts/proteins in the core dataset that mapped to KEGG pathways were classified as success items in the population (M). The proteins in each pathway were designated as the sample (n) and the proteins in the core dataset that mapped to the pathway (i). The hypergeometric probability (*P* value) of a particular pathway was calculated based on following formula: 

$$\text{P}=1-\sum_{i=0}^{m-1}\frac{\left(\begin{array}{c} M\\ i \end{array}\right)\left(\begin{array}{c} N-M\\ n-i \end{array}\right)}{\left(\begin{array}{c} N\\ n \end{array}\right)}$$

We chose a cut off value of 0.05 (P<0.05) for statistical significance.

### Phylogenetic Analysis

The candidate sequences from *Latrodectus tredecimguttatus* are used as baits to search homologoue genes by BLAST against Uniprot database (e-value < 1e^5^). Only the best match homologues of each species were used for further study. Multiple sequence alignments were executed by ClustalX 2.1 in a slow model. Phylogenetic analyses were conducted by MAGA 5.2 using Maximum Likelihood algorithm with 1000 bootstrap replicates. 

### Searching toxin-like proteins

Three strategies were used to identify toxin-like proteins, sequence homology searching, domain architecture prediction and cysteine-pattern alignment. A BLAST search against the Uniprot database identified 70 toxin-like proteins that were homologues of known toxins. Comparison of the domain architecture of all proteins with that of known toxins led to the identification of 20 additional toxin candidates. Cysteine rich domains, which are characteristic of spider toxins, were also used as key features to detect potential toxins [39]. First, we extracted 284 cysteine-rich structure patterns from 38,323 known toxins in the ATDB database [57] and ArachnoServer [58] and used them to search the core dataset. Finally, 10 high confidence toxin sequences containing 7 different cysteine patterns were identified manually. Domain architectures were predicted by the SMART and Pfam servers (http://smart.embl.de and http://pfam.janelia.org)[59,60]. All candidate sequences are execute multiple sequence alignments were executed by ClustalX 2.1 in a slow model.

## Supporting Information

File S1 **Figure S1, The distribution of EST sequences in different GO categories.** **Figure S2, Distribution of the length of identified transcripts**. **Figure S3, Scatter plots of RPKM distribution of genes in the KEGG classes**. **Figure S4, The mapping of identified transcripts/proteins (marked as red) in the spliceosome pathway of KEGG database**. **Figure S5, The mapping of identified transcripts /proteins (marked as red) in the pathway of protein process in endoplasmic reticulum in KEGG database**. **Figure S6, Sequence characteristics of members in orphan families**. Sequence characteristics of members in orphan families. The potential toxins, which haven’t homologue’s function annotations, are classified into orphan families including two groups: one comprises toxins predicted from Cys patterns, and the other is based on sequence homology with known toxins containing domains. Within Cys patterns, the char “#” represents any three amino acids other than Cys. For other toxins, the domain architectures were predicted by the SMART and Pfam servers [59,60]. The character “-F” appended to protein ID numbers indicates that these sequences are fragments but not full-length proteins. The abbreviations of domain names are as follow: EGF (SMART ID: SM00181); KU (SMART ID: SM00131), glyco_hydro_56 (Pfam ID: PF01630); crust_neurohorm (Pfam ID: PF01147). **Figure S7, The abundance of toxin families in different functional categories**. Bars represent toxin families clustered based on their functional characteristics. The sum of RPKM values for each class and category are labeled. Neurotoxins including the ANK superfamily, the SCP family and the lycotoxin family; Assistant toxins including theriditoxin family; Proteases including ctenitoxin family; Function unknown toxins including scorpion toxin like family and the orphan family. **Figure S8, Phylogenomic trees for trypsin, scorpion toxin-like, lycotoxin, ctenitoxin, SCP family**. Phylogenomic trees of trypsin, scorpion toxin-like, lycotoxin, ctenitoxin and SCP families. A. Ctenitoxin family; B. Trypsin family; C. Scorpion toxin-like family; D. SCP family; E. Lycotoxin family. The members of family and their homologues from other spiders are colored as blue and red on branches. For spider species that have transcriptomic data were highlighted by a green line. **Figure S9, Phylogenetic tree of ANK superfamily toxins and their homologues from other 45 species**. Phylogenetic tree of ANK superfamily toxins and their homologues from other 45 species. Color code: pink for α-LTX-Lt1a family1; blue for α-LTX-Lt1a family2; green for δ-LIT-Lt1a family; red for α-LIT-Lt1a family; brown for ANK family. All phylogeny analyses are performed with MEGA 5.2 using Maximum Likelihood algorithm and 1000 bootstrap tests. The numbers on the branches are the supporting percentages of 1000 bootstrap tests. **Table S1, RPKM distribution in the top ten of three GO namespaces**. **Table S2, RPKM statistics of Ion channel in *Latrodectustredecimguttatus***. **Table S3, The statistics of RPKM in KEGG pathway superclass**. **Table S4, The RPKM list of sub-classes of the “Genetic information processing” category in KEGG database**. **Table S5, List of toxins identified by sequence analyses**. **Table S6, Known ion channel toxins in five venomous species**. **Table S7, Full names/abbreviations’ and taxonomic classification of 18 species in phylogenetic analysis**. **Table S8, Full names/abbreviations and taxonomic classification of 54 arthropod species**. **Table S9, Full names/abbreviations and taxonomic classification of species shown in Figure S8**.
(PDF)
