# Supplementary material for: The Venom Gland Transcriptome of Latrodectus tredecimguttatus Revealed by Deep Sequencing and cDNA Library Analysis
Source: PLoS One. 2013 Nov 28;8(11):e81357. doi: 10.1371/journal.pone.0081357 (PMC3842942; doi:10.1371/journal.pone.0081357)
Supplement: File S1 — Figure S1, The distribution of EST sequences in different GO categories. Figure S2, Distribution of the length of identified transcripts. Figure S3, Scatter plots of RPKM distribution of genes in the KEGG classes. Figure S4, The mapping of identified transcripts/proteins (marked as red) in the spliceosome pathway of KEGG database. Figure S5, The mapping of identified transcripts /proteins (marked as red) in the pathway of protein process in endoplasmic reticulum in KEGG database. Figure S6, Sequence characteristics of members in orphan families. Sequence characteristics of members in orphan families. The potential toxins, which haven’t homologue’s function annotations, are classified into orphan families including two groups: one comprises toxins predicted from Cys patterns, and the other is based on sequence homology with known toxins containing domains. Within Cys patterns, the char “#” represents any three amino acids other than Cys. For other toxins, the domain architectures were predicted by the SMART and Pfam servers [59,60]. The character “-F” appended to protein ID numbers indicates that these sequences are fragments but not full-length proteins. The abbreviations of domain names are as follow: EGF (SMART ID: SM00181); KU (SMART ID: SM00131), glyco_hydro_56 (Pfam ID: PF01630); crust_neurohorm (Pfam ID: PF01147). Figure S7, The abundance of toxin families in different functional categories. Bars represent toxin families clustered based on their functional characteristics. The sum of RPKM values for each class and category are labeled. Neurotoxins including the ANK superfamily, the SCP family and the lycotoxin family; Assistant toxins including theriditoxin family; Proteases including ctenitoxin family; Function unknown toxins including scorpion toxin like family and the orphan family. Figure S8, Phylogenomic trees for trypsin, scorpion toxin-like, lycotoxin, ctenitoxin, SCP family. Phylogenomic trees of trypsin, scorpion toxin-like, lycotoxin, ctenitoxin and [file pone.0081357.s001.pdf]

## Legends for File S1

**Figure S1: The distribution of EST sequences in different GO categories**

**Figure S2: Distribution of the length of identified transcripts**

**Figure S3: Scatter plots of RPKM distribution of genes in the KEGG classes.**

**Figure S4: The mapping of identified transcripts/proteins (marked as red) in the spliceosome pathway of KEGG database**

**Figure S5: The mapping of identified transcripts /proteins (marked as red) in the pathway of protein process in endoplasmic reticulum in KEGG database.**

**Figure S6: Sequence characteristics of members in orphan families.** Sequence characteristics of members in orphan families. The potential toxins, which haven't homologue's function annotations, are classified into orphan families including two groups: one comprises toxins predicted from Cys patterns, and the other is based on sequence homology with known toxins containing domains. Within Cys patterns, the char "#" represents any three amino acids other than Cys. For other toxins, the domain architectures were predicted by the SMART and Pfam servers [59,60]. The character "-F" appended to protein ID numbers indicates that these sequences are fragments but not full-length proteins. The abbreviations of domain names are as follow: EGF (SMART ID: SM00181); KU (SMART ID: SM00131), glyco_hydro_56 (Pfam ID: PF01630); crust_neurohorm (Pfam ID: PF01147).

**Figure S7: The abundance of toxin families in different functional categories.** Bars represent toxin families clustered based on their functional characteristics. The sum of RPKM values for each class and category are labeled. Neurotoxins including the ANK superfamily, the SCP family and the lycotoxin family; Assistant toxins including theriditoxin family; Proteases including ctenitoxin family; Function unknown toxins including scorpion toxin like family and the orphan family.

**Figure S8: Phylogenomic trees for trypsin, scorpion toxin-like, lycotoxin, ctenitoxin, SCP family.** Phylogenomic trees of trypsin, scorpion toxin-like, lycotoxin, ctenitoxin and SCP families. A. Ctenitoxin family; B. Trypsin family; C. Scorpion toxin-like family; D. SCP family; E. Lycotoxin family. The members of family and their homologues from other spiders are colored as blue and red on branches. For spider species that have transcriptomic data were highlighted by a green line.

**Figure S9: Phylogenetic tree of ANK superfamily toxins and their homologues from other 45 species.** Phylogenetic tree of ANK superfamily toxins and their homologues from other 45 species. Color code: pink for α-LTX-Lt1a family1; blue for α-LTX-Lt1a family2; green for δ-LIT-Lt1a family; red for α-LIT-Lt1a family; brown for ANK family. All phylogeny analyses are performed with MEGA 5.2 using Maximum Likelihood algorithm and 1000 bootstrap tests. The numbers on the branches are the supporting percentages of 1000 bootstrap tests.

**Table S1: RPKM distribution in the top ten of three GO namespaces.**

**Table S2: RPKM statistics of Ion channel in *Latrodectus tredecimguttatus*.**

**Table S3: The statistics of RPKM in KEGG pathway superclass.**

**Table S4: The RPKM list of sub-classes of the "Genetic information processing" category in KEGG database.**

**Table S5: List of toxins identified by sequence analyses.**

**Table S6: Known ion channel toxins in five venomous species.**

**Table S7: Full names/abbreviations' and taxonomic classification of 18 species in phylogenetic analysis.**

**Table S8: Full names/abbreviations and taxonomic classification of 54 arthropod species.**

**Table S9: Full names/abbreviations and taxonomic classification of species shown in Figure S8.**

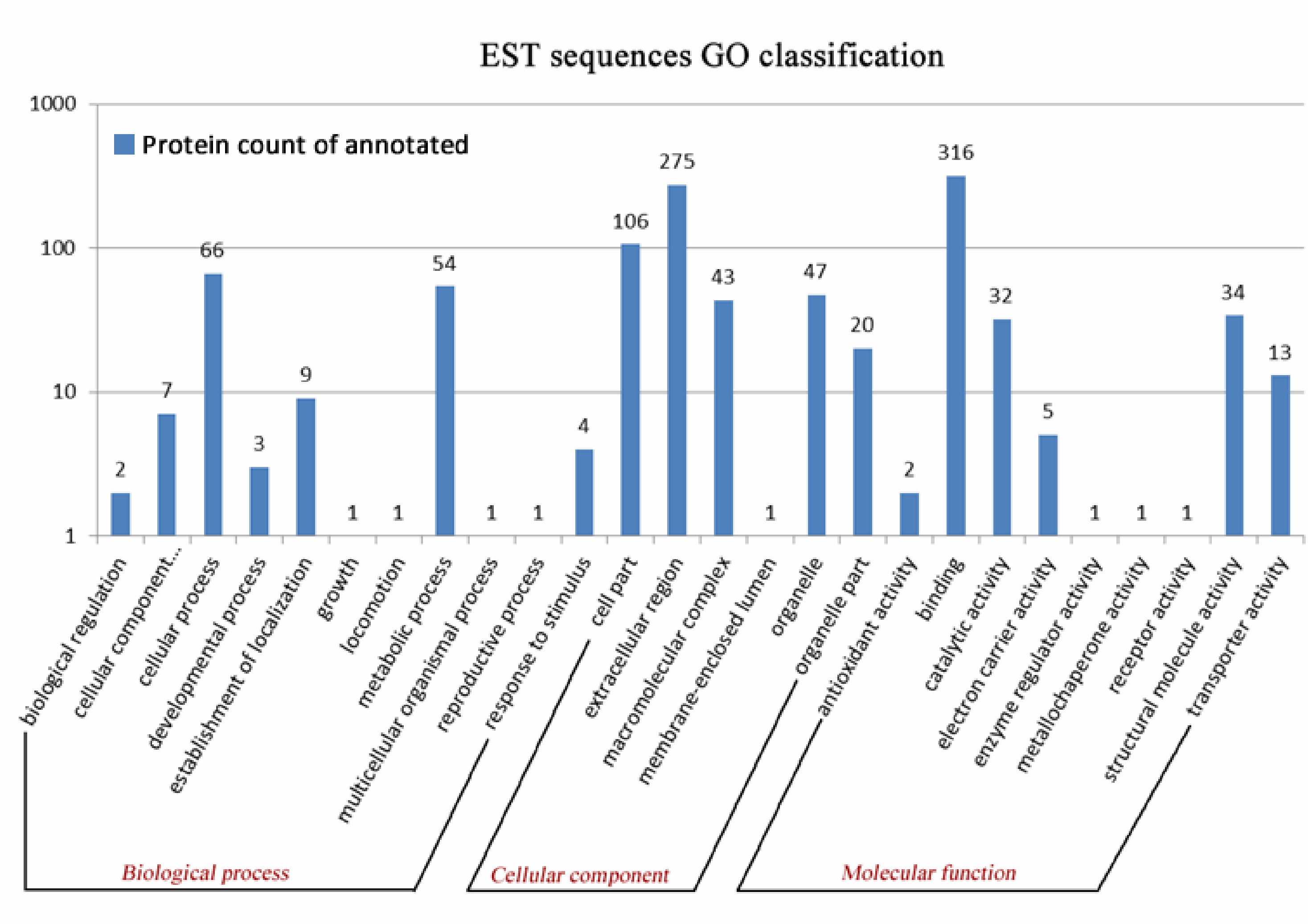
EST sequences GO classification
Protein count of annotated
1000
100
10
1
2
7
66
3
9
1
1
54
1
1
4
106
275
43
1
47
20
2
316
32
5
1
1
1
34
13
biological regulation
cellular component...
cellular process
developmental process
establishment of localization
growth
locomotion
metabolic process
multicellular organismal process
reproductive process
response to stimulus
cell part
extracellular region
macromolecular complex
membrane-enclosed lumen
organelle
organelle part
antioxidant activity
binding
catalytic activity
electron carrier activity
enzyme regulator activity
metallochaperone activity
receptor activity
structural molecule activity
transporter activity
Biological process
Cellular component
Molecular function

**Figure S1**

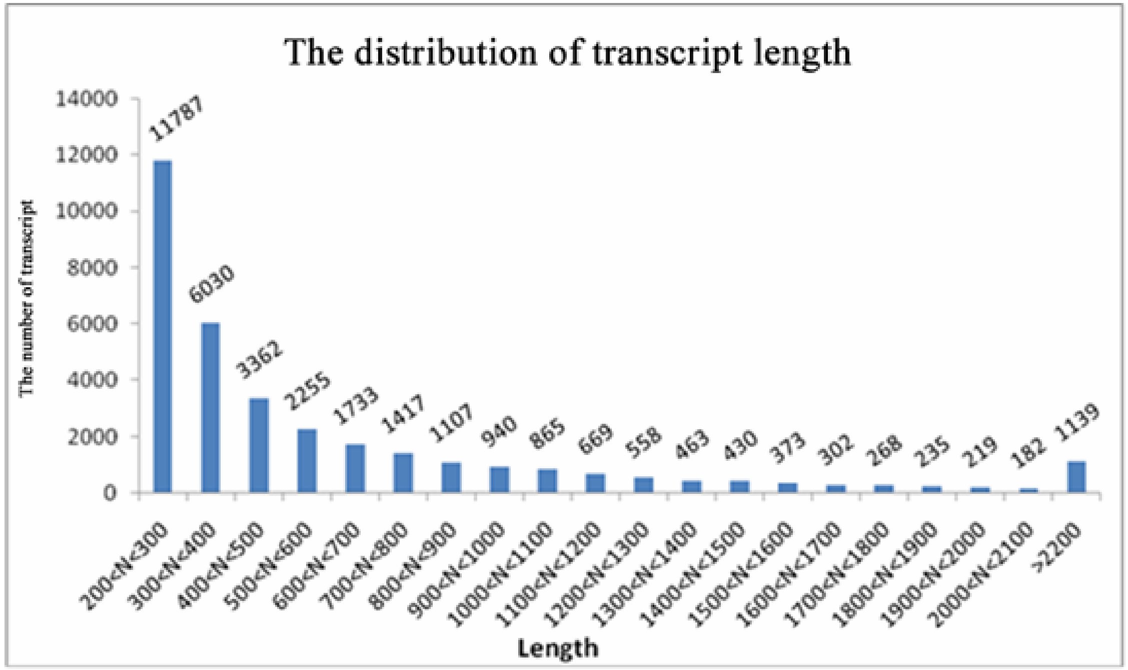

The distribution of transcript length
The number of transcript
14000
12000
10000
8000
6000
4000
2000
0
11787
6030
3362
2255
1733
1417
1107
940
865
669
558
463
430
373
302
268
235
219
182
1139
200<N<300
300<N<400
400<N<500
500<N<600
600<N<700
700<N<800
800<N<900
900<N<1000
1000<N<1100
1100<N<1200
1200<N<1300
1300<N<1400
1400<N<1500
1500<N<1600
1600<N<1700
1700<N<1800
1800<N<1900
1900<N<2000
2000<N<2100
>2200
Length


**Figure S2**

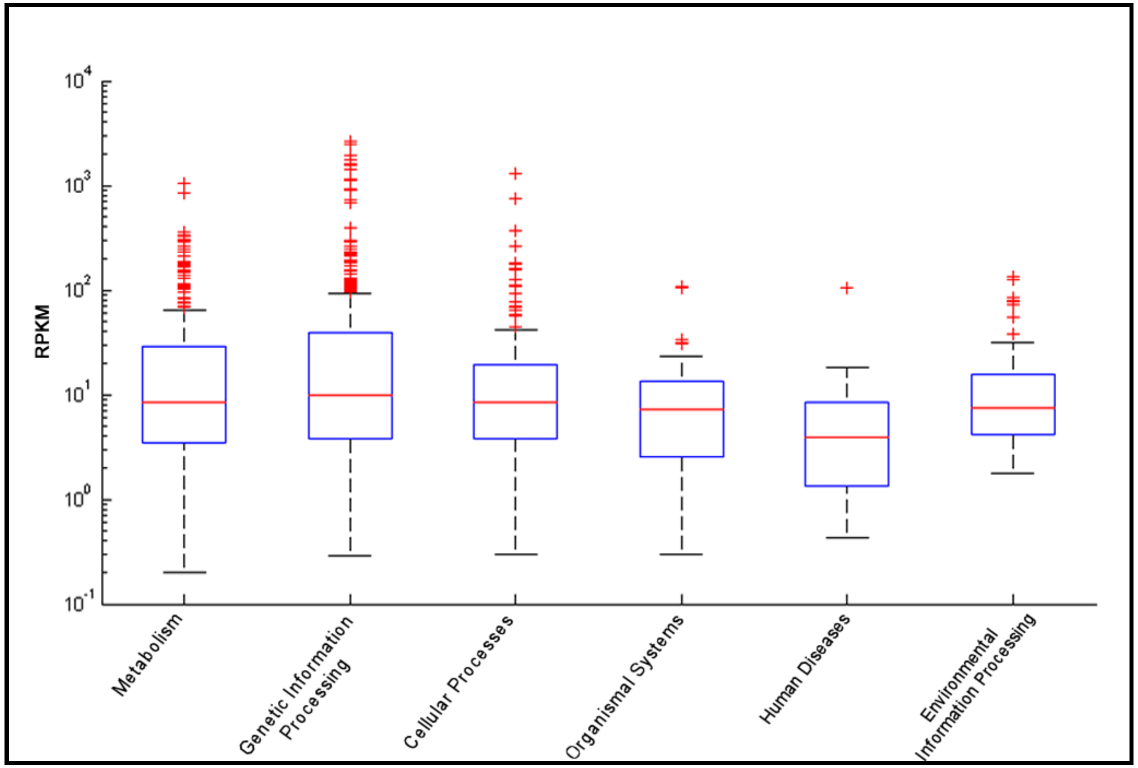
RPKM
$10^4$
$10^3$
$10^2$
$10^1$
$10^0$
$10^{-1}$
Metabolism
Genetic Information Processing
Cellular Processes
Organismal Systems
Human Diseases
Environmental Information Processing

**Figure S3**

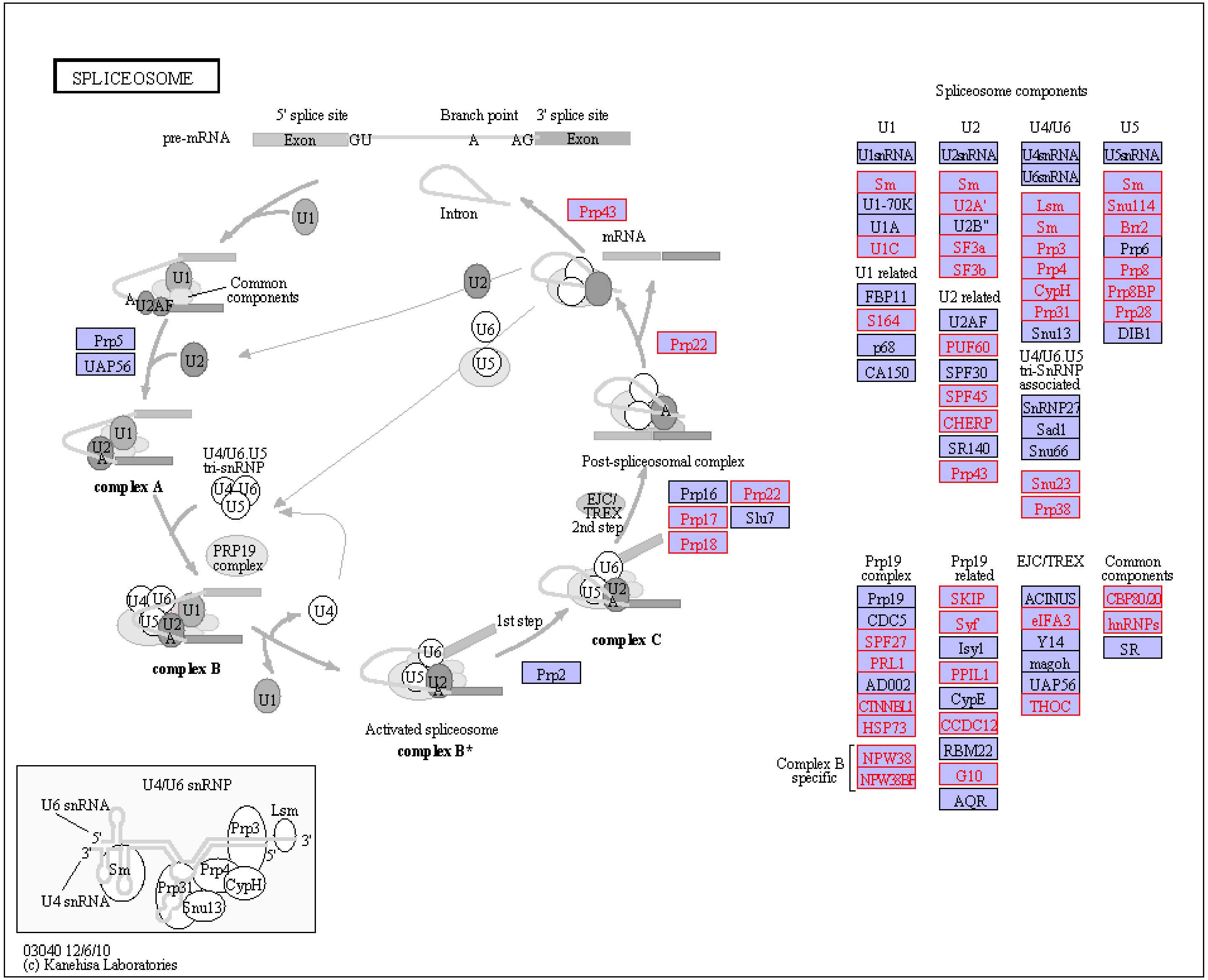
SPLICEOSOME
pre-mRNA
5' splice site
Branch point
3' splice site
Exon
GU
A
AG
Exon
Intron
mRNA
Prp43
Common components
U2AF
Prp5
UAP56
complex A
U4/U6.U5 tri-snRNP
PRP19 complex
complex B
Activated spliceosome
complex B*
Prp2
1st step
complex C
EJC/TREX
2nd step
Prp16
Prp22
Prp17
Slu7
Prp18
Post-spliceosomal complex
Prp22
U4/U6 snRNP
U6 snRNA
U4 snRNA
Lsm
Prp3
Prp4
CypH
Prp31
Snu13
Sm
03040 12/6/10
(c) Kanehisa Laboratories
Spliceosome components
U1
U2
U4/U6
U5
U1snRNA
Sm
U1-70K
U1A
U1C
U1 related
FBP11
S164
p68
CA150
U2snRNA
Sm
U2A'
U2B"
SF3a
SF3b
U2 related
U2AF
PUF60
SPF30
SPF45
CHERP
SR140
Prp43
U4snRNA
U6snRNA
Lsm
Sm
Prp3
Prp4
CypH
Prp31
Snu13
U4/U6.U5 tri-SnRNP associated
SnRNP27
Sad1
Snu66
Snu23
Prp38
U5snRNA
Sm
Snu114
Brr2
Prp6
Prp8
Prp8BP
Prp28
DIB1
Prp19 complex
Prp19
CDC5
SPF27
PRL1
AD002
CTNNBL1
HSP73
Complex B specific
NPW38
NPW38BP
Prp19 related
SKIP
Syf
Isy1
PPIL1
CypE
CCDC12
RBM22
G10
AQR
EJC/TREX
ACINUS
eIFA3
Y14
magoh
UAP56
THOC
Common components
CBP80/20
hnRNPs
SR

**Figure S4**

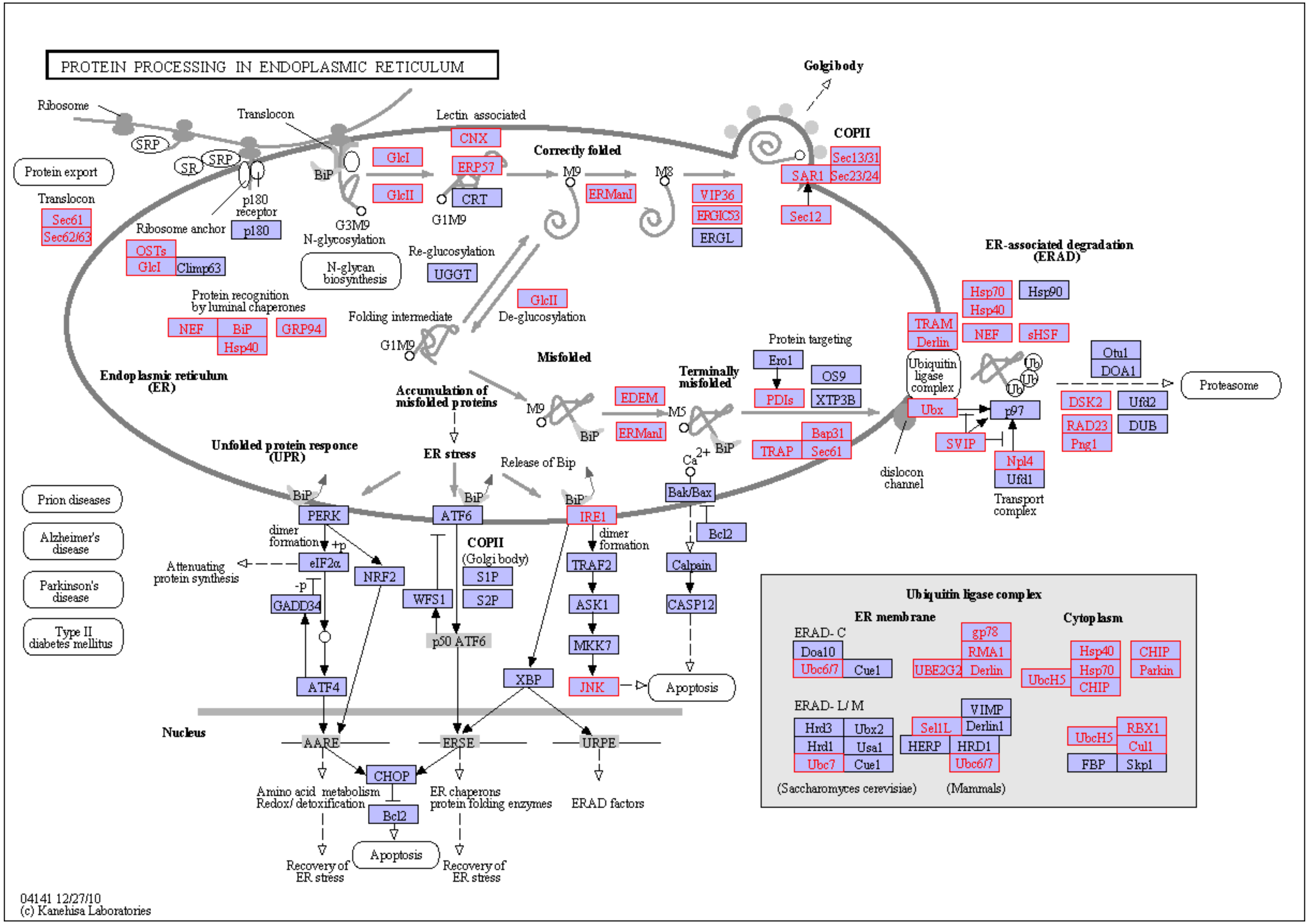
PROTEIN PROCESSING IN ENDOPLASMIC RETICULUM
Ribosome
Translocon
Lectin associated
Golgi body
COPII
Protein export
SRP
SR
BiP
p180 receptor
Ribosome anchor
p180
Translocon
Sec61
Sec62/63
OSTs
GlcI
Climp63
GlcII
CNX
ERP57
CRT
G3M9
N-glycosylation
G1M9
Correctly folded
M9
M8
ERManI
VIP36
ERGIC53
ERGL
SAR1
Sec13/31
Sec23/24
Sec12
N-glycan biosynthesis
Re-glucosylation
UGGT
De-glucosylation
Folding intermediate
Protein recognition by luminal chaperones
NEF
GRP94
Hsp40
Endoplasmic reticulum (ER)
Misfolded
Accumulation of misfolded proteins
EDEM
M5
Terminally misfolded
Protein targeting
Ero1
PDIs
OS9
XTP3B
Bap31
TRAP
Ca2+
ER-associated degradation (ERAD)
Hsp70
Hsp90
TRAM
Derlin
sHSF
Ubiquitin ligase complex
Ub
Otu1
DOA1
Proteasome
Ubx
p97
DSK2
Ufd2
RAD23
DUB
Png1
SVIP
Npl4
Ufd1
Transport complex
dislocon channel
Unfolded protein responce (UPR)
ER stress
Release of Bip
Prion diseases
Alzheimer's disease
Parkinson's disease
Type II diabetes mellitus
PERK
ATF6
IRE1
Bak/Bax
Bcl2
dimer formation
+p
eIF2α
Attenuating protein synthesis
-p
NRF2
GADD34
WFS1
COPII (Golgi body)
S1P
S2P
p50 ATF6
TRAF2
ASK1
MKK7
JNK
Calpain
CASP12
Apoptosis
XBP
ATF4
Nucleus
AARE
ERSE
URPE
CHOP
Amino acid metabolism Redox/ detoxification
ER chaperons protein folding enzymes
ERAD factors
Recovery of ER stress
Ubiquitin ligase complex
ER membrane
Cytoplasm
ERAD- C
Doa10
Ubc6/7
Cue1
gp78
RMA1
UBE2G2
Derlin
UbcH5
CHIP
Parkin
ERAD- L/ M
Hrd3
Ubx2
Hrd1
Usa1
Ubc7
VIMP
SelIL
Derlin1
HERP
HRD1
RBX1
Cul1
FBP
Skp1
(Saccharomyces cerevisiae)
(Mammals)
04141 12/27/10
(c) Kanehisa Laboratories

**Figure S5**

Orphan Family

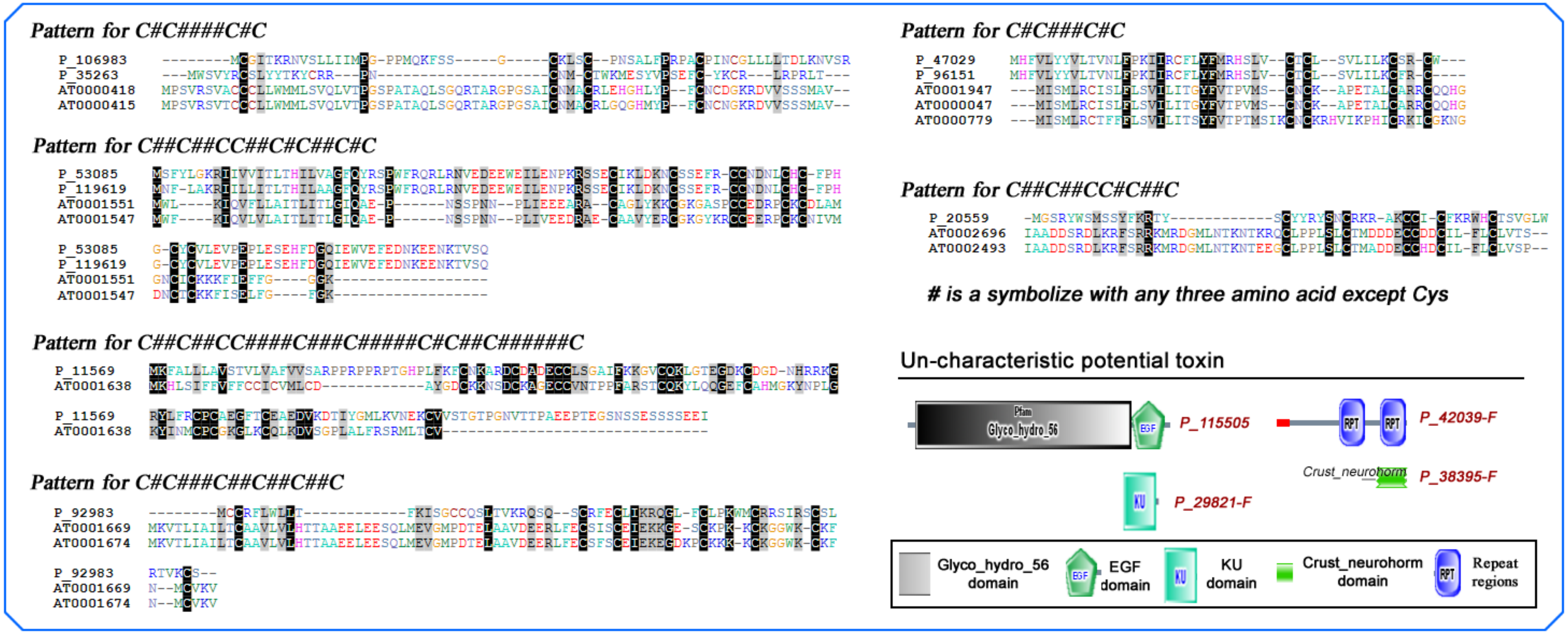


Figure S6

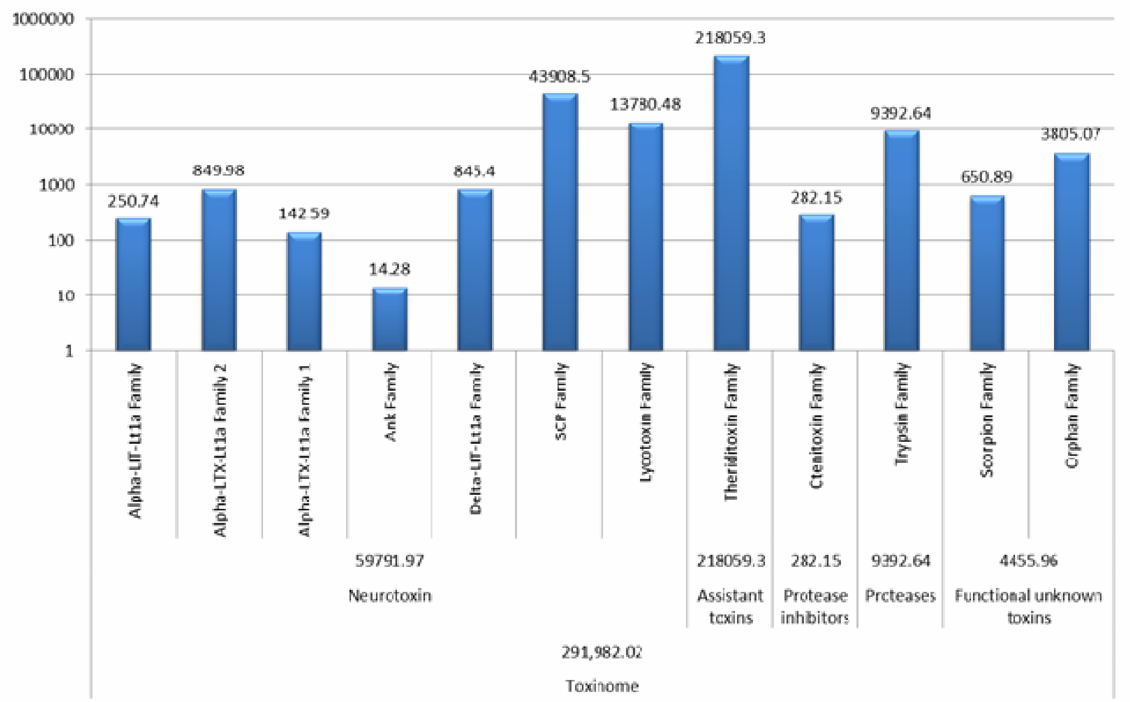

1000000
100000
10000
1000
100
10
1
250.74
849.98
142.59
14.28
845.4
43908.5
13780.48
218059.3
282.15
9392.64
650.89
3805.07
Alpha-LIT-Lt1a Family
Alpha-LTX-Lt1a Family 2
Alpha-LTX-Lt1a Family 1
Ank Family
Delta-LIT-Lt1a Family
SCP Family
Lycotoxin Family
Theriditoxin Family
Ctenitoxin Family
Trypsin Family
Scorpion Family
Orphan Family
59791.97
Neurotoxin
218059.3
Assistant toxins
282.15
Protease inhibitors
9392.64
Proteases
4455.96
Functional unknown toxins
291,982.02
Toxinome


**Figure S7**

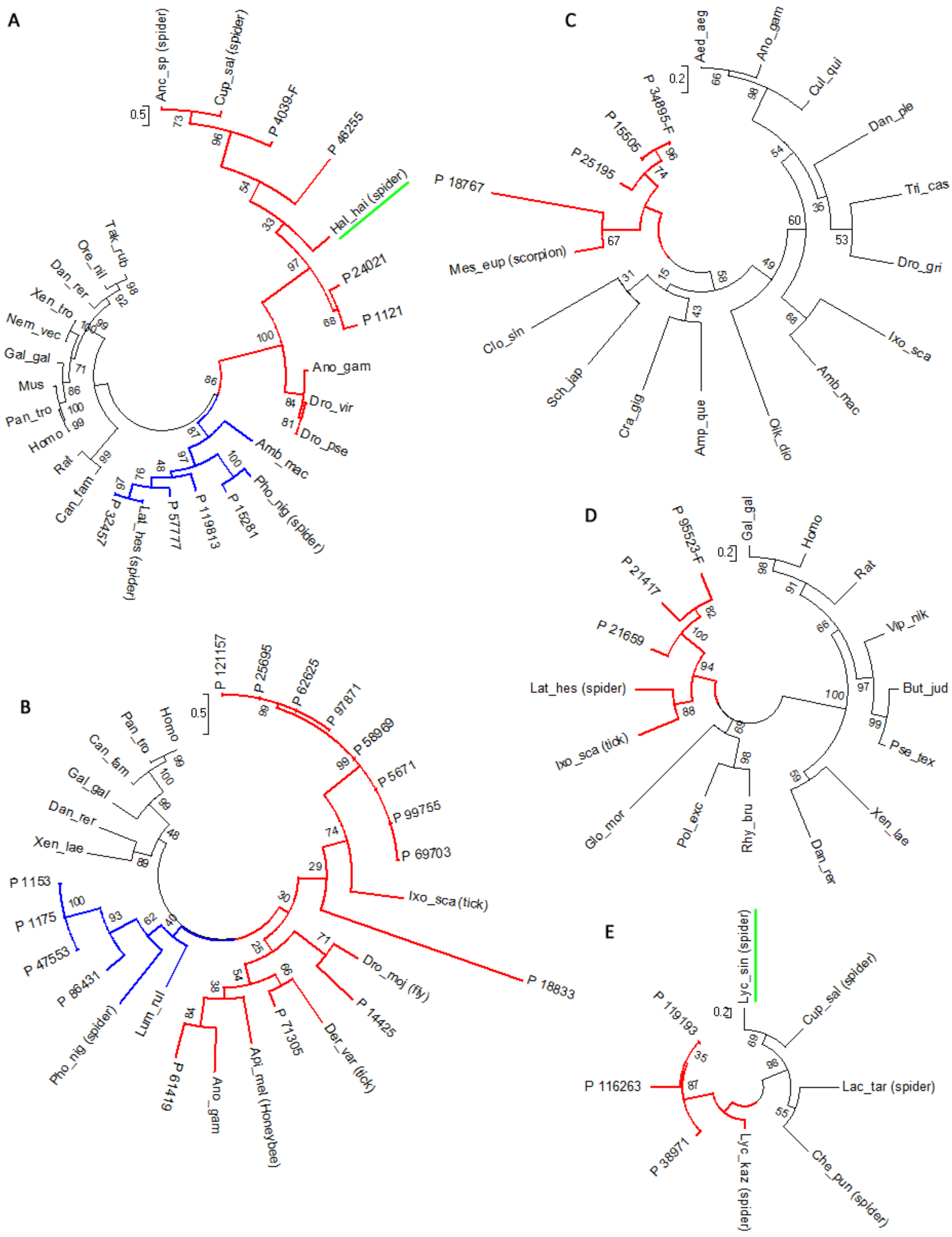
A
0.5
Anc_sp (spider)
Cup_sal (spider)
P 4039-F
P 46255
Hal_hal (spider)
P 24021
P 1121
Ano_gam
Dro_vir
Dro_pse
Amb_mac
Pho_nig (spider)
P 15281
P 119813
P 57777
Lat_hes (spider)
P 32457
Can_fam
Rat
Homo
Pan_tro
Mus
Gal_gal
Nem_vec
Xen_tro
Dan_rer
Ore_nil
Tak_rub
B
0.5
P 121157
P 25695
P 62625
P 97871
P 58969
P 5671
P 99755
P 69703
Ixo_sca (tick)
P 18833
Dro_moj (fly)
P 14425
Der_var (tick)
P 71305
Api_mel (Honeybee)
Ano_gam
P 61419
Lum_rul
Pho_nig (spider)
P 86431
P 47553
P 1175
P 1153
Xen_lae
Dan_rer
Gal_gal
Can_fam
Pan_tro
Homo
C
0.2
Aed_aeg
Ano_gam
Cul_qui
Dan_ple
Tri_cas
Dro_gri
Ixo_sca
Amb_mac
Oik_dio
Amp_que
Cra_gig
Sch_jap
Clo_sin
Mes_eup (scorpion)
P 18767
P 25195
P 15505
P 34895-F
D
0.2
Gal_gal
Homo
Rat
Vip_nik
But_jud
Pse_tex
Xen_lae
Dan_rer
Rhy_bru
Pol_exc
Glo_mor
Ixo_sca (tick)
Lat_hes (spider)
P 21659
P 21417
P 95523-F
E
0.2
Lyc_sin (spider)
Cup_sal (spider)
Lac_tar (spider)
Che_pun (spider)
Lyc_kaz (spider)
P 38971
P 116263
P 119193

**Figure S8**

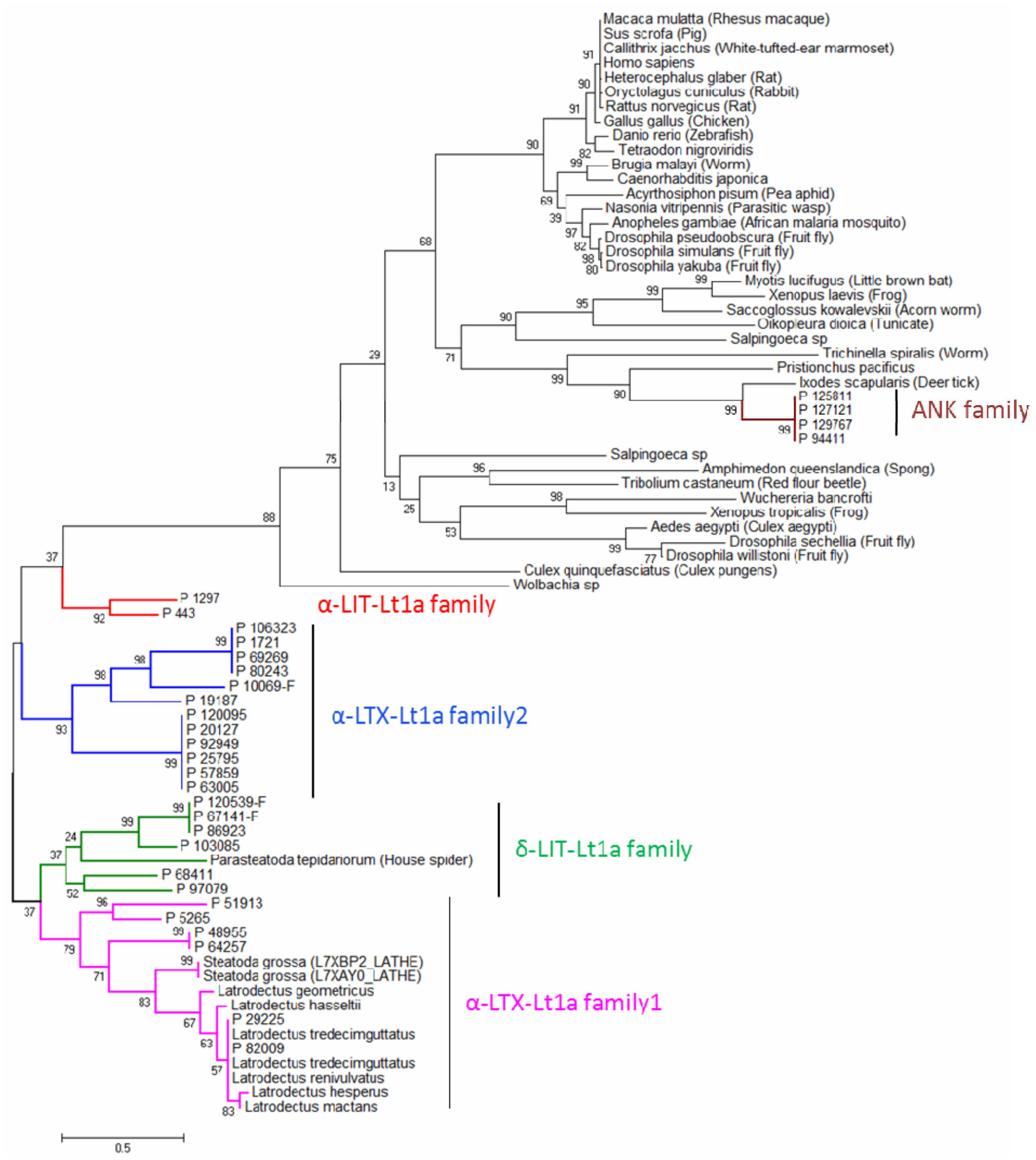

Macaca mulatta (Rhesus macaque)
Sus scrofa (Pig)
Callithrix jacchus (White-tufted-ear marmoset)
Homo sapiens
Heterocephalus glaber (Rat)
Oryctolagus cuniculus (Rabbit)
Rattus norvegicus (Rat)
Gallus gallus (Chicken)
Danio rerio (Zebrafish)
Tetraodon nigroviridis
Brugia malayi (Worm)
Caenorhabditis japonica
Acyrthosiphon pisum (Pea aphid)
Nasonia vitripennis (Parasitic wasp)
Anopheles gambiae (African malaria mosquito)
Drosophila pseudoobscura (Fruit fly)
Drosophila simulans (Fruit fly)
Drosophila yakuba (Fruit fly)
Myotis lucifugus (Little brown bat)
Xenopus laevis (Frog)
Saccoglossus kowalevskii (Acorn worm)
Oikopleura dioica (Tunicate)
Salpingoeca sp
Trichinella spiralis (Worm)
Pristionchus pacificus
Ixodes scapularis (Deer tick)
P 125811
P 127121
P 129767
P 94411
ANK family
Salpingoeca sp
Amphimedon queenslandica (Spong)
Tribolium castaneum (Red flour beetle)
Wuchereria bancrofti
Xenopus tropicalis (Frog)
Aedes aegypti (Culex aegypti)
Drosophila sechellia (Fruit fly)
Drosophila willistoni (Fruit fly)
Culex quinquefasciatus (Culex pungens)
Wolbachia sp
P 1297
P 443
α-LIT-Lt1a family
P 106323
P 1721
P 69269
P 80243
P 10069-F
P 19187
P 120095
P 20127
P 92949
P 25795
P 57859
P 63005
α-LTX-Lt1a family2
P 120539-F
P 67141-F
P 86923
P 103085
Parasteatoda tepidariorum (House spider)
P 68411
P 97079
δ-LIT-Lt1a family
P 51913
P 5265
P 48955
P 64257
Steatoda grossa (L7XBP2_LATHE)
Steatoda grossa (L7XAY0_LATHE)
Latrodectus geometricus
Latrodectus hasseltii
P 29225
Latrodectus tredecimguttatus
P 82009
Latrodectus tredecimguttatus
Latrodectus renivulvatus
Latrodectus hesperus
Latrodectus mactans
α-LTX-Lt1a family1
0.5


**Figure S9**

**Table S1.** RPKM distribution in the top ten GO items in three GO namespaces

| Biological process namespace | | | Cellular component namespace | | | Molecular function namespace | | |
|---|---|---|---|---|---|---|---|---|
| **GO Item** | **Count** | **RPKM** | **GO Item** | **Count** | **RPKM** | **GO Item** | **Count** | **RPKM** |
| Translation | 222 | 101254.5 | Extracellular region | 69 | 87229.42 | Neuropeptide hormone activity | 4 | 218072.3 |
| Proteolysis | 183 | 29747.34 | Ribosome | 202 | 99301.79 | Structural constituent of ribosome | 214 | 106616 |
| Electron transport chain | 6 | 11005.62 | Integral to membrane | 617 | 54733.82 | ATP binding | 622 | 24843.5 |
| Transmembrane transport | 96 | 10427.08 | Cytoplasm | 272 | 23632.59 | Metalloendopeptidase activity | 48 | 17527.55 |
| ATP synthesis coupled proton transport | 16 | 10399.12 | Mitochondrial inner membrane | 29 | 17699 | Oxygen transporter activity | 16 | 17100.38 |
| Transport | 47 | 7432.82 | Respiratory chain | 6 | 12817.73 | Electron carrier activity | 82 | 16007.03 |
| Aerobic respiration | 1 | 6966.76 | Small ribosomal subunit | 16 | 8400.76 | Calcium ion binding | 158 | 15046.09 |
| Chitin metabolic process | 12 | 5714.95 | Eukaryotic translation elongation factor 1 complex | 7 | 8236.08 | Oxidoreductase activity | 117 | 14029.13 |
| Translational elongation | 9 | 5654.64 | Mitochondrion | 36 | 8214.48 | Binding | 333 | 13876.24 |
| GTP biosynthetic process | 5 | 5213.21 | Membrane | 239 | 7967.85 | RNA binding | 182 | 13527.49 |

**Table S2.** RPKM statistics of ion channels in *Latrodectus tredecimguttatus*

| Class Name | Ion channel Name | Attacked by spider toxin | RPKM | RPKM Count |
|---|---|---|---|---|
| **Cation Channel** | Novel protein similar to vertebrate calcium channel voltage-dependent alpha 2/delta subunit 2 (CACNA2D2) | Unknown | 4.13 | ***162.86*** |
| | Potassium channel protein putative | Unknown | 12.08 | |
| | Potassium channel putative | Unknown | 2.26 | |
| | Regulator of ATP-sensitive K+ channels putative | Unknown | 91.89 | |
| | Cyclic nucleotide-gated cation channel | Unknown | 1.05 | |
| | Transient receptor potential cation channel protein 4 | Unknown | 1.45 | |
| | Trimeric intracellular cation channel type A putative | Unknown | 40.27 | |
| | Voltage-dependent calcium channel subunit alpha-2/delta-3 | Unknown | 4.68 | |
| | Inward rectifier K+ channel putative | Unknown | 2.54 | |
| | Voltage-gated hydrogen channel putative | Unknown | 2.51 | |
| **Anion Channel** | Chloride channel putative | Unknown | 10 | ***970.65*** |
| | Chloride channel protein 3 | Unknown | 4.26 | |
| | Glutamate gated chloride channel | Unknown | 6.23 | |
| | pH-sensitive chloride channel | Unknown | 4.16 | |
| | Voltage-dependent anion-selective channel putative | Unknown | 946 | |
| **Other** | Potassium intermediate/small conductance calcium-activated channel subfamily N member 2 | Unknown | 1.05 | ***6.12*** |
| | Cyclic nucleotide-gated ion channel LCNG1 | Unknown | 5.07 | |

**Table S3.** The statistics of RPKMs in KEGG pathway superclass

| KEGG pathway superclass | Pathway Number | RPKM (%) | Protein Number | Average RPKM |
|---|---|---|---|---|
| Genetic Information Processing | 21 | 32142.33 (48%) | 522 | 61.57 |
| Metabolism | 75 | 19924.51 (30%) | 588 | 33.88 |
| Cellular Processes | 11 | 6168.96 (9%) | 161 | 38.31 |
| Human Diseases | 21 | 5182.66 (8%) | 40 | 129.56 |
| Environmental Information Processing | 14 | 3086.34 (4%) | 86 | 133.27 |
| Organismal Systems | 22 | 764.83 (1%) | 58 | 13.18 |

**Table S4.** The RPKM list of sub-classes of the "Genetic information processing" category in KEGG database

| **Sub-class Name** | **RPKM** |
|---|---|
| Ribosome | 11166.9 |
| Protein processing in endoplasmic reticulum | 5193.12 |
| RNA transport | 4974.49 |
| Protein export | 2689.95 |
| Proteasome | 1720.84 |
| Spliceosome | 1183.85 |
| Ubiquitin mediated proteolysis | 1168.3 |
| Ribosome biogenesis in eukaryotes | 712.82 |
| mRNA surveillance pathway | 581.5 |
| Aminoacyl-tRNA biosynthesis | 421.24 |
| Nucleotide excision repair | 412.8 |
| RNA polymerase | 397.21 |
| RNA degradation | 373.73 |
| Basal transcription factors | 327.53 |
| Homologous recombinatio | 303.85 |
| SNARE interactions in vesicular transport | 257.33 |
| DNA replication | 79.05 |
| Mismatch repair | 67.74 |
| Base excision repair | 53.79 |
| Sulfur relay system | 47.58 |
| Non-homologous end-joining | 8.71 |

**Table S5:** List of toxins identified by sequence analyses

| Family Name | Homologue/Conserved Domain | Protein ID | RPKM |
|---|---|---|---|
| **Alpha-LIT-Lt1a Family** | Homologue: Alpha-LCT-Lt1a | P_443 | 250.74 |
| | Homologue: Alpha-LIT-Lt1a | P_1297 | |
| **Theriditoxin Family** | Homologue: Alpha-latrotoxin-associated LMWP2 | P_66861 | 218059.3 |
| | Homologue: Alpha-latrotoxin-associated LMWP | P_89055 | |
| **Alpha-LTX-Lt1a Family 2** | Homologue: Alpha-LTX-Lt1a | P_51913, P_29225, P_124863-F, P_64257, P_48955, P_82009, P_5265 | 849.98 |
| **Alpha-LTX-Lt1a Family 1** | Homologue: Alpha-LTX-Lt1a | P_120095, P_25795, P_1721, P_57859, P_20127, P_63005, P_92949, P_19187, P_10069-F, P_69269, P_106323, P_80243 | 142.59 |
| **Ank Family** | Domain matching | P_63979-F, P_77051, P_36165-F, P_73235, P_97059, P_87955, P_116129, P_127121, P_129767, P_125811, P_94411, P_77485 | 153.24 |
| **SCP Family** | Homologue: Cysteine-rich secretory protein | P_21417, P_21659, P_95523-F, P_67749 | 44017.56 |
| **Delta-LIT-Lt1a Family** | Homologue: Delta-LIT-Lt1a | P_67141-F, P_97079, P_103085, P_86923, P_68411, P_120539-F | 845.4 |
| **Ctenitoxin Family** | Domain matching | P_46255 | 282.15 |
| | Homologue: U24-ctenitoxin-Pn1a | P_32457, P_15281, P_119813, P_57777 | |
| | Homologue: U3-ctenitoxin-Asp1a | P_4039-F | |
| | Homologue: U9-ctenitoxin-Pr1a | P_24021, P_86613-F | |
| | Homologue: U19-ctenitoxin-Pn1a | P_1121 | |
| **Trypsin Family** | Domain matching | P_5671, P_121157, P_99755, P_18833, P_14425, P_61419, P_25695, P_97871, P_62625, P_58969, P_69703, P_71305, P_1153 | 9392.64 |
| | Homologue: Fibrinolytic enzyme | P_1175 | |
| | Homologue: Trypsin-7 | P_47553 | |
| | Homologue: U21-ctenitoxin-Pn1a | P_86431 | |
| **Lycotoxin Family** | Homologue: U1-aranetoxin-Av1a | P_29821-F | 13781.7 |
| | Homologue: U1-lycotoxin-Ls1hh | P_116263 | |
| | Homologue: U4-lycotoxin-Ls1a | P_119193, P_38971 | |
| **Scorpion Family** | Homologue: Venom protein-2 | P_18767 | 650.89 |
| | Homologue: Venom toxin-like peptide-6 | P_15505, P_34895-F, P_25195 | |
| **Orphan Family** | Scaffold matching | P_92983 , P_96151, P_106983, P_35263, P_47029, P_11569, P_53085, P_119619, P_20559 | 3816.79 |
| | Homologue: Orcokinin-like peptide | P_42039-F | |
| | Homologue: Ion transport peptide | P_38395-F | |
| | Homologue: Venom hyaluronidase | P_115505 | |

*Note*, known toxins are colored by blue; Toxins and toxin families that were firstly reported in are colored by red and green, respectively

**Table S6**. Known ion channel toxins in five venom species

| | Sodium channel | Potassium channel | Calcium channel |
|---|---|---|---|
| Scorpion | 263 | 188 | 47 |
| Snake | 20 | 32 | 50 |
| Spider | 188 | 62 | 101 |
| Sea anemone | 67 | 29 | 0 |
| Cone | 108 | 39 | 76 |

**Table S7.** Full names/abbreviations' and taxonomic classification of 18 species

| | |
|---|---|
| **Amphibia** | Xenopus tropicalis (Western clawed frog) [Xen_tro] |
| **Arthropoda** | Anopheles gambiae (African malaria mosquito) [Ano_gam] |
| | Apis mellifera (Honeybee) [Api_mel] |
| | Drosophila melanogaster (Fruit fly) [Dro_mel] |
| | Latrodectus tredecimguttatus (Lat_tre) |
| **Aves** | Gallus gallus (Chicken) [Gal] |
| **Fungi** | Dictyostelium discoideum (Slime mold) [Dic_dis] |
| | Dictyostelium fasciculatum (strain SH3) (Slime mold) [Dic_fas] |
| **Mammalia** | Canis familiaris (Dog) (Canis lupus familiaris) [Can_fam] |
| | Mus musculus (Mouse) [Mus] |
| | Pan troglodytes (Chimpanzee) [Pan_tro] |
| | Homo sapiens (Human) [Homo] |
| | Pan troglodytes (Chimpanzee)[Pan_tro] |
| | Rattus norvegicus (Rat) [Rat] |
| **Nematoda** | Caenorhabditis elegans [Cae_ele] |
| **Teleostei** | Danio rerio (Zebrafish) (Brachydanio rerio) [Dan_rer] |
| | Takifugu rubripes (Japanese pufferfish) (Fugu rubripes) [Tak_rub] |
| **Other** | Ciona intestinalis (Transparent sea squirt) [Cio_int] |

.

**Table S8.** Full names/abbreviations and taxonomic classification of 54 arthropod species

| Lineage | | | | Species |
|---|---|---|---|---|
| **Arthropoda** | **Mandibulata** | **Pancrustacea** | **Hexapoda** | Metajapyx subterraneus (Jap); Eumesocampa frigilis (Efr); Orchesella imitari (Oim); Ischnura verticalis(Ive); Plathemis lydia (Lly); Hexagenia limbata (May); Periplaneta americana (Pam); Acheta domesticus (Ado); Nicoletia meinerti (Nme); Ctenolepisma lineata (Cli); Pedetontus saltator (Psa); Machiloides banksi (Mba); Ephemerella inconstans (Ein); |
| | | | **Crustacea** | Neogonodactylus oerstedii (Neo); Libinia emarginata (Lem); Armadillidium vulgare (Avu3); Eurytemora affinis (Eaf); Acanthocyclops vernalis (A369); Daphnia magna (Dma); Lynceus sp. (Lyn); Artemia salina (Asa3); Streptocephalus seali (ufs); Loxothylacus texanus (Lox); Semibalanus balanoides (Bba); Chthamalus fragilis (Cfr); Lepas anserifera (Lean) |
| | | | **Oligostraca** | Skogsbergia lerneri (Skle); Argulus sp. (Arg2); Armillifer armillatus (Aar) |
| | | **Myriapoda** | | Scutigera coleoptrata (Scol); Eurypauropus spinosus (Eury); Craterostigmus tasmanianus (Ctas2); Hanseniella sp.(Han2); Scutigerella sp.(Scu3); Polyxenus fasciculatus (Pol2); Abacion magnum (Ama); Polyzonium germanicum (Pge2); |
| | **Chelicerata** | | **Arachnida & Merostomata** | Prokoenenia wheeleri (Pwh); Dinothrombium pandorae (Din2); Leiobunum verrucosum (Lnig); Amblyomma sp. (Amb2); Idiogaryops pumilis (Ipum); Carcinoscorpius rotundicauda (Cro2); Cryptocellus centralis (Crp2); Eremocosta gigasella (Egig); Hadrurus arizonensis (Hari); Heterometrus spinifer (Hsp); Aphonopelma chalcodes (Ach); Phrynus marginemaculatus (Pma2); Stenochrus portoricensis (Stp); Latrodectus tredecimguttatus (Lat_tre) |
| | | | **Pycnogonida** | Achelia echinata (Aeli); Endeis laevis (Ele); Ammothea hilgendorfi (Ahi) |

**Table S9.** Full names/abbreviations and taxonomic classification of species shown in Figure S8.

| | |
|---|---|
| **Amphibia** | Xenopus tropicalis (Xen_tro) |
| **Arthropoda** | Anopheles gambiae (Ano_gam), Apis mellifera (Api_mel), Drosophila melanogaster (Dro_mel), Drosophila pseudoobscura pseudoobscura (Dro_pse), Amblyomma maculatum (Amb_mac), Haplopelma hainanum (Hap_hai), Phoneutria nigriventer (Pho_nig), Cupiennius salei (Cup_sal), Ancylometes sp (Anc_sp), Latrodectus hesperus (Lat_hes), Lycosa singoriensis (Lyc_sin), Lachesana tarabaevi (Lac_tar), Cheiracanthium punctorium (Che_pun), Lycosa kazakhstanicus (Lyc_kaz), Aedes aegypti(Aed_aeg), Tribolium castaneum(Tri_cas), Drosophila grimshawi(Dro_gri), Danaus plexippus(Dan_ple), Anopheles gambiae str (Ano_gam), Culex quinquefasciatus(Cul_qui), Ixodes scapularis(Ixo_sca), Amblyomma maculatum(Amb_mac), Crassostrea gigas(Cra_gig), Mesobuthus eupeus(Mes_eup), Buthotus judaicus(But_jud), Glossina morsitans morsitans (Glo_mor), Rhynchium brunneum (Rhy_bru), Polistes exclamans(Pol_exc) |
| **Aves** | Gallus gallus (Gal) |
| **Mammalia** | Pan troglodytes (Chimpanzee), Mus musculus (Mus), Pan troglodytes (Chimpanzee), Rattus norvegicus (Rat), Homo sapiens (Homo), Canis familiaris (Dog) |
| **Trematoda** | Schistosoma japonicum(Sch_jap), Clonorchis sinensis(Clo_sin) |
| **Teleostei** | Danio rerio (Dan_rer)[ Zebrafish], Oreochromis niloticus (Ore_nil), Takifugu rubripes (Tak_rub) |
| **Other** | Nematostella vectensis (Nem_vec), Oikopleura dioica(Oik_dio), Amphimedon queenslandica (Amp_gue), Pseudonaja textilis(Pse_tex), Vipera nikolskii(Vip_nik) |
